# Succinate promotes stem cell migration through the GPR91-dependent regulation of DRP1-mediated mitochondrial fission

**DOI:** 10.1038/s41598-017-12692-x

**Published:** 2017-10-03

**Authors:** So Hee Ko, Gee Euhn Choi, Ji Young Oh, Hyun Jik Lee, Jun Sung Kim, Chang Woo Chae, Diana Choi, Ho Jae Han

**Affiliations:** 1 Department of Veterinary Physiology, College of Veterinary Medicine, Research Institute for Veterinary Science, and BK21 PLUS program for Creative Veterinary Research Center, Seoul National University, Seoul, 08826 South Korea; 2 Department of Agricultural Biotechnology, Animal Biotechnology Major, and Research Institute for Agriculture and Life science, Seoul National University, Seoul, 08826 South Korea; 3 Department of Biological Sciences, Mount Holyoke College, South Hadley, Massachusetts 01075 USA

## Abstract

The role of metabolites produced from stem cell metabolism has been emerged as signaling molecules to regulate stem cell behaviors such as migration. The mitochondrial morphology is closely associated with the metabolic balance and stem cell function. However, the physiological role of succinate on human mesenchymal stem cell (hMSC) migration by regulating the mitochondrial morphology remains unclear. Here, we investigate the effect of succinate on hMSC migration via regulation of mitochondrial dynamics and its related signaling pathway. Succinate (50 μM) significantly accelerates hMSC migration. Succinate increases phosphorylation of pan-PKC, especially the atypical PKCζ level which was blocked by the knockdown of Gα_q_ and Gα_12._ Activated PKCζ subsequently phosphorylates p38 MAPK. Cytosolic DRP1 is phosphorylated by p38 MAPK and results in DRP1 translocation to the mitochondria outer membrane, eventually inducing mitochondrial fragmentation. Mitochondrial fission-induced mitochondrial function elevates mitochondrial ROS (mtROS) levels and activates Rho GTPases, which then induces F-actin formation. Furthermore, in a skin excisional wound model, we found the effects of succinate-pretreated hMSC enhanced wound closure, vascularization and re-epithelialization and confirmed that DRP1 has a vital role in injured tissue regeneration. Overall, succinate promotes DRP1-mediated mitochondrial fission via GPR91, consequently stimulating the hMSC migration through mtROS-induced F-actin formation.

## Introduction

Stem cell metabolism delicately regulates the balance of the metabolic needs associated with the cellular state through aerobic glycolysis, glutamine catabolism, and *de novo* synthesis of fatty acid and lipids^1–3^. Recently, TCA cycle metabolites such as fumarate and succinate have been widely reported to play an important role in maintaining stem cell function by adjusting their cellular ratios^4,5^. In addition to the metabolites that are used for cellular energy production, they role in cell-to-cell communication and sensing microenvironmental conditions to adapt^4^. Interestingly, metabolic intermediates have recently been found to have novel signaling functions, with the potential to adjust stem cell behavior with regard to stem cell niche^5,6^. Stem cell niche, as most hypoxia, is closely associated with general metabolic stress that is considered to be a mediator of hypoxia-induced succinate accumulation^7,8^. Furthermore, succinate has been reported to regulate the stem cell functions under the cellular physiologic condition^9–11^. Accordingly, succinate was reported not only to be the central metabolites in the cellular energy metabolism but also to regulate cell functions by initiating the signaling cascade. Extracellular succinate binds to a G-protein coupled receptor that triggers signal transduction to initiate physiological response such as cell division, capillary formation, and migration^11–15^. Thus, the comprehensive roles of succinate and its specific receptor in stem cells need to be investigated as a bioactive molecule involved in the activation of stem cell functions facilitating tissue regeneration during the wound repair process. Although this area of research is burgeoning, still numerous limitations remain, including our incomplete understanding of stem cell metabolite that has functions both *in vitro* and *in vivo*. Hence, the comprehensive roles of succinate and those mechanisms that regulates stem cell physiology remain to be investigated^16,17^.

Mitochondria are well known organelles that tightly maintain cellular homeostasis by balancing energy production during which metabolites can accumulate. Mitochondria are widespread around the cytosol, regulating various physiological functions of the cell^18^. To adapt to a new metabolic milieu, mitochondria continuously change their morphology through the fusion and fission cycle, reciprocally responding to the metabolic alteration^19–21^. Mitochondrial dynamics are involved in various cellular functions such as redox regulation and cell proliferation^22^. Mitochondrial fission is mostly mediated by GTPase activity on dynamin-related protein 1 (DRP1), and consequently, DRP1 translocates from the cytosol to the mitochondrial outer membrane region. On the other hand, mitochondrial fusion is mediated mainly by the optic atrophy 1 (OPA1) located on the mitochondrial outer membrane and mitofusion 1/2 (MFN1/2) located on the inner membrane^23^. Although the importance of balancing the mitochondrial morphology through mitochondrial dynamics is now understood, there is little knowledge about the mitochondrial dynamics involved in stem cell migration. Many studies have shown that mitochondrial fission is observed in regions with high levels of energy demand, such as the axon growth axis and during oocyte maturation and cell migration^24,25^. The shape and activities of proteins related to mitochondrial dynamics are mediated by post-translational modifications such as phosphorylation. Thus, efforts have been made to discover the upstream regulator of mitochondrial dynamics proteins, highlighting the emerging significance of mitochondria in stem cells^26^.

Human mesenchymal stem cells (hMSCs) have emerged in clinical applications due to their ability to differentiate into multi-lineage cell types such as chondrocytes, osteoblasts, and adipocytes^27^. hMSCs have been widely used due to their ability to self-renewal and less immunogenicity compared to other stem cells, as well as financial advantages, availability and less ethical concerns compared to embryonic stem cells^28,29^. For these reasons, understanding and manipulating the metabolic regulations of hMSC can provide a strategy for enhancing beneficial properties and safety of the clinical applications of hMSC. The present study aims to investigate the effects of succinate on hMSC mitochondrial dynamics and its related signaling pathways in hMSC function, along with the clinical applications in skin wound healing *in vivo*.

## Results

### Succinate induces hMSC migration through GPR91-mediated PKC activation

**Figure 1:**
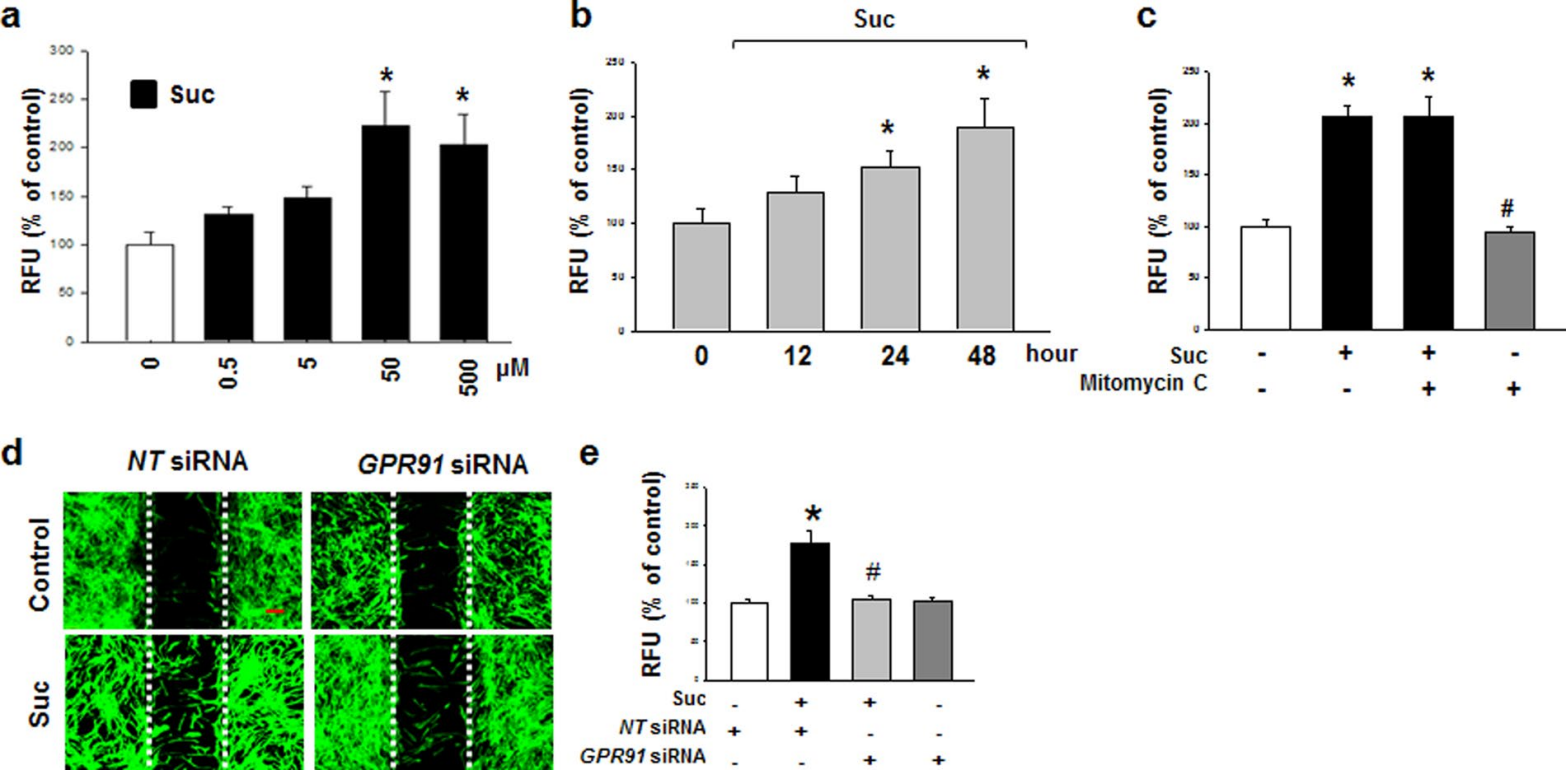
Effect of succinate on hMSC migration. **(a)** Dose responses of succinate in Oris cell migration assay were shown. Data represent the mean ± SEM. n = 4, ^*^p < *0*.*05* versus control. **(b)** Time responses of succinate in Oris™ cell migration assay were shown. Data represent the mean ± SEM. n = 4. ^*^p < *0*.*05* versus control. **(c)** Effect of mitomycin C in succinate-induced hMSC migration. Cells were treated with mitomycin C (1 μg/ml) for 90 min before succinate exposure. Data represent the mean ± SEM. n = 4. ^*^p < *0*.*01* versus control. ^*#*^*p* < *0*.*05* versus succinate alone. **(d)** To determine whether GPR91 involved in the succinate-induced hMSC migration, *GPR91* siRNA were transfected. Cells were stained with phalloidin AlexaFlour 488 (Green) to identify the migrated cells. Scale bar = 50 μm, magnification; ×80. **(e)** hMSC migration quantified with Oris cell migration assay was shown. Data indicate means ± SEM. n = 4, ^*^p < *0*.*05* versus *NT* siRNA transfected cells, ^#^*p* < *0*.*01* versus succinate with *NT* siRNA transfected sample. Abbreviations: RFU, relative fluorescence units.

Because the dose range of succinate that stimulates the cell function varies on the cell types, we tested the effect of succinate on hMSC motility in a dose and time dependent manner. hMSCs were treated with various concentrations (0–500 μM) for 24 hr, and it was found that 50 to 500 μM of succinate increased stem cell migration (Fig. 1a). Stem cell motility was significantly increased after 24 to 48 hr succinate treatment (Fig. 1b). Additionally, we treated mitomycin C (1 μg/mL) for 90 min prior to the exposure to succinate, which inhibits the cell cycle, but did not affect succinate-induced stem cell motility, indicating that the effect of succinate is independent of hMSC proliferation (Fig. 1c). Succinate-induced hMSC migration into the wound area was inhibited by *GPR91* siRNA transfection, indicating that succinate-induced hMSCs migration was mediated by GPR91 signaling pathway (Fig. 1d). We carried out an Oris migration assay to quantify the succinate-induced stem cell migration, and *GPR91* siRNA attenuated succinate-induced hMSC migration (Fig. 1e).

**Figure 2:**
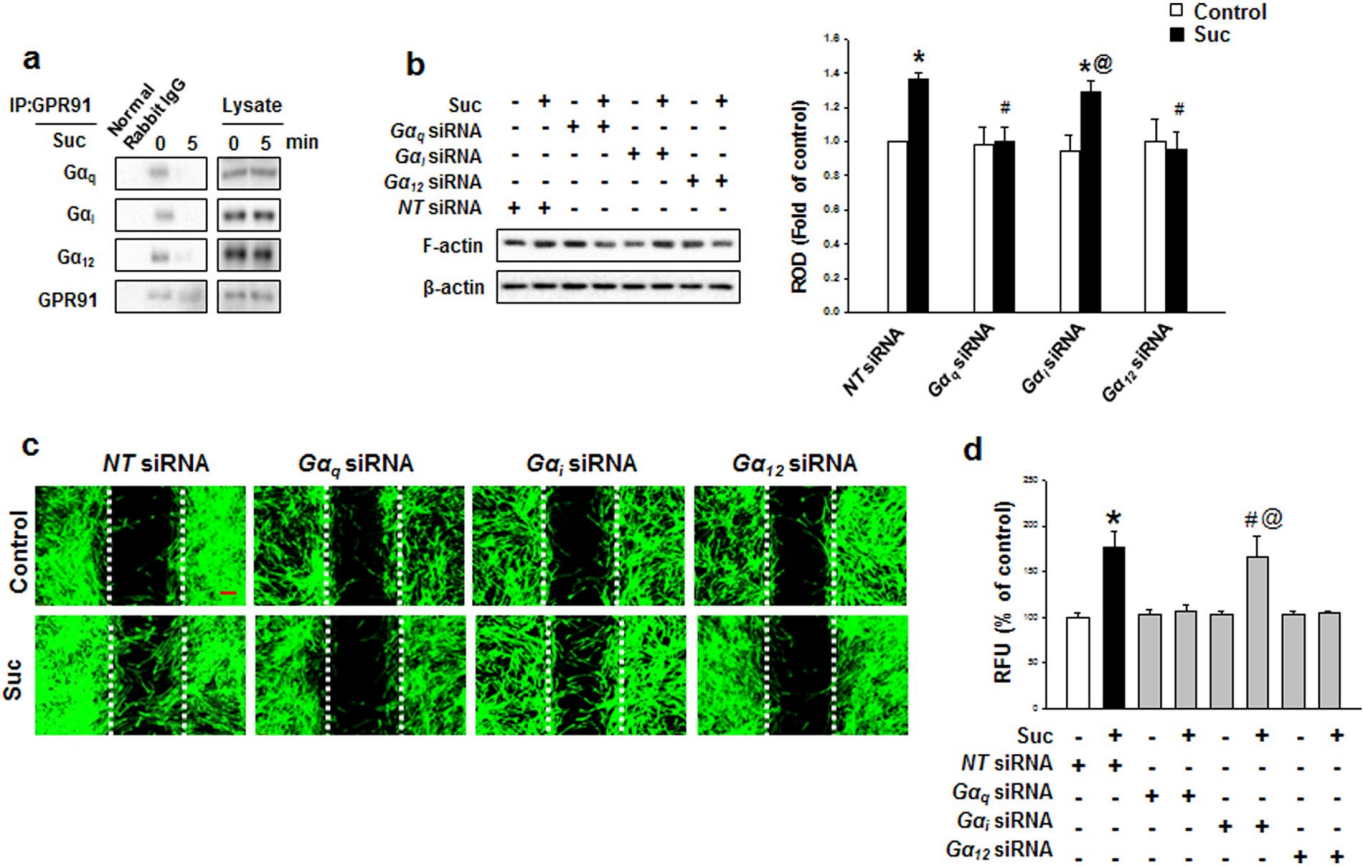
Involvements of GPR91 and and Gα complex in succinate-induced hMSC migration. **(a)** hMSC were treated with succinate for 5 min. GPR91 co-immunoprecipitated with Gα_q_, Gα_i_, and Gα_12_ and the level of each protein is shown. **(b)** *Gα*_*q*_, *Gα*_*i*_, and *Gα*_*12*_ siRNA transfected to hMSC prior to treatment of succinate for 24 hrs. Cells were harvested for western blotting using anti-F-actin antibody. Data represent the mean ± SEM. n = 3. ^*^p < *0*.*01* versus control, ^*#*^*p* < *0*.*05* versus succinate treated cell, ^*@*^*p* < *0*.*05* versus *Gα*_*i*_ siRNA alone. **(c)** Cells were transfected with *Gα complex* siRNA prior to exposure to succinate and then Wound healing assay was performed to identify succinate-induced cell migration. Scale bar = 50 μm, magnification; ×80. **(d)** *Gα complex* siRNA transfected hMSC migration was quantified with Oris™ cell migration assay. Data represent the mean ± SEM. n = 3, ^*^p < *0*.*05* versus control, ^*#*^*p* < *0*.*01* versus succinate alone, ^*@*^*p* < *0*.*01* versus *Gα*_*i*_ siRNA alone. Abbreviations: RFU, relative fluorescence units; ROD, relative optical density.

**Figure 3:**
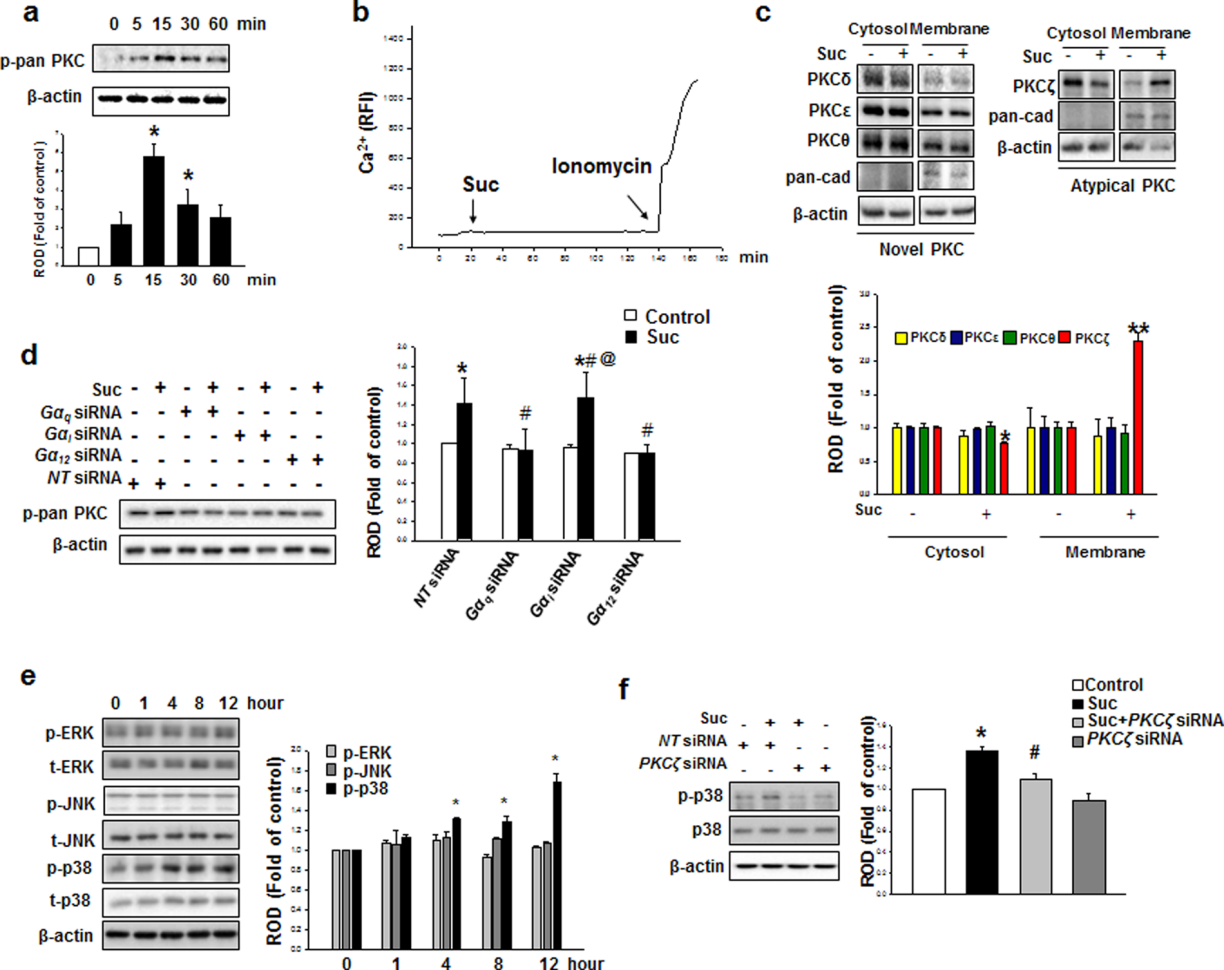
Succinate-induced PKC phosphorylation involved in p38 MAPK activation. **(a)** The cells were incubated with succinate (50 μM) and then harvested. Total protein was analyzed by western blotting with phospho PKC antibodies. Data represent the mean ± SEM. n = 3, ^*^p < *0*.*05* versus control. **(b)** Cells were stained with Fluo-3AM (2 mM) for 40 min and treated with succinate. Ionomycin (10 μM) was used as positive control. The changes of Fluo-3AM fluorescence were represented in relative fluorescence intensity (RFI, F/F_0_%, arbitrary unit). **(c)** Cells were treated with succinate for 15 min and harvested for cytosolic/membrane fractionation. Translocation of PKC was detected with novel PKC and atypical PKC antibodies. Data represent the mean ± SEM. n = 3. ^*^p < *0*.*05* versus cytosolic control. ^**^p < *0*.*05* versus membrane part control. **(d)** Cells were transfected with *Gα*_*q*_, *Gα*_*i*_, and *Gα*_*12*_ siRNA prior to succinate exposure for 15 min and then harvested for western blot analysis with detecting p-pan PKC. Data represent the mean ± SEM. n = 3, ^*^p < *0*.*05* versus control, ^*#*^*p* < *0*.*05* versus hMSC with succinate, ^*@*^*p* < *0*.*05* versus *Gα*_*i*_ siRNA alone. **(e)** hMSCs were treated with succinate and then harvested for western blotting to show the phosphorylation of MAPKs. Data represent the mean ± SEM. n = 4, ^*^p < *0*.*05* versus 0 time. **(f)** Cells were transfected with *PKCζ* siRNA prior to succinate exposure for 12 hr and then western blotting to show the phosphorylation of p38 MAPK. Data represent the mean ± SEM. n = 3, ^*^p < *0*.*05* versus control, ^*#*^*p* < *0*.*05* versus hMSC with succinate. Abbreviations: ROD, relative optical density.

After treatment with 50 μM of succinate for 24 hr, GPR91 protein expression levels were analyzed with western blotting. GPR91 protein expression increased, suggesting that succinate initiates hMSC signaling cascade through GPR91 receptor (Supplementary Fig. S1a and b). Succinate led to the detachment of Gα_q_, Gα_i_, and Gα_12_ subunits from GPR91, indicating that the all Gα complexes were activated after succinate treatment (Fig. 2a). We need to determine which subunits played functional roles in succinate-induced hMSC migration. To determine which Gα subunits were involved in succinate-induced hMSC migration, *Gα subunit* siRNA transfection was performed, which revealed that Gα_q_ and Gα_12_ subunits were involved in F-actin reorganization (Fig. 2b). Consistently, inhibition of Gα_q_ and Gα_12_ by siRNA transfection suppressed the succinate-induced cell migration in both *in vitro* wound healing assay and Oris cell migration assay (Fig. 2c and d). These results showed that Gα_q_ and Gα_12_ activation of GPR91 complex have a functional role in hMSC migration. Studies have reported that succinate activates GPR91 to regulate PLC signaling, which induces IP_3_ accumulation and thus resulting in calcium mobilization and PKC activation^14^. This motivated us to assess the level of PKC phosphorylation and the maximum phospho-pan PKC level was observed after 15 min of succinate treatment (Fig. 3a). To examine whether increment of succinate-induced PKC phosphorylation levels was due to Ca^2+^ influx, Ca^2+^ was stained with Fluo-3AM for 40 min. There was no change in the level of Ca^2+^ influx after succinate treatment, while ionomycin (Ca^2+^ ionophore), as a positive control, drastically elevated Ca^2+^ influx (Fig. 3b). Since Ca^2+^ was not involved in the phosphorylation of PKC, we examined Ca^2+^-independent PKC expression in the Novel PKC (PKCδ, PKCε, and PKCθ) and the Atypical PKC (PKCζ) groups. Among Ca^2+^-independent PKC samples, we observed PKCζ alone translocated from cytosol to membrane after succinate treatment (Fig. 3c). The knockdown of Gα complex was found to attenuate succinate-induced PKC phosphorylation, indicating that phosphorylation of PKC also depended on the regulation of Gα_q_ and Gα_12_ (Fig. 3d). To investigate the succinate effect through GPR91 even more precisely, we examined mitogen activated protein kinase (MAPK) signaling pathway, which is known to have a role in GPR91 downstream kinase cascade. Among MAPKs, phosphorylation levels of p38 MAPK increased in a time-dependent manner, especially 4 hr after succinate treatment (Fig. 3e). To examine whether PKC activation is involved in the succinate-induced p38 MAPK activation, we transfected *PKCζ* siRNA prior to the exposure to succinate for 12 hr, which resulted in the inhibition of succinate-induced p38 MAPK phosphorylation^30^ (Fig. 3f). Taken together, succinate activates GPR91/PKC ζ/p38 MAPK signaling pathway of hMSC motility.

### Succinate-induced p38 activation by PKC involved in DRP1 phosphorylation

**Figure 4:**
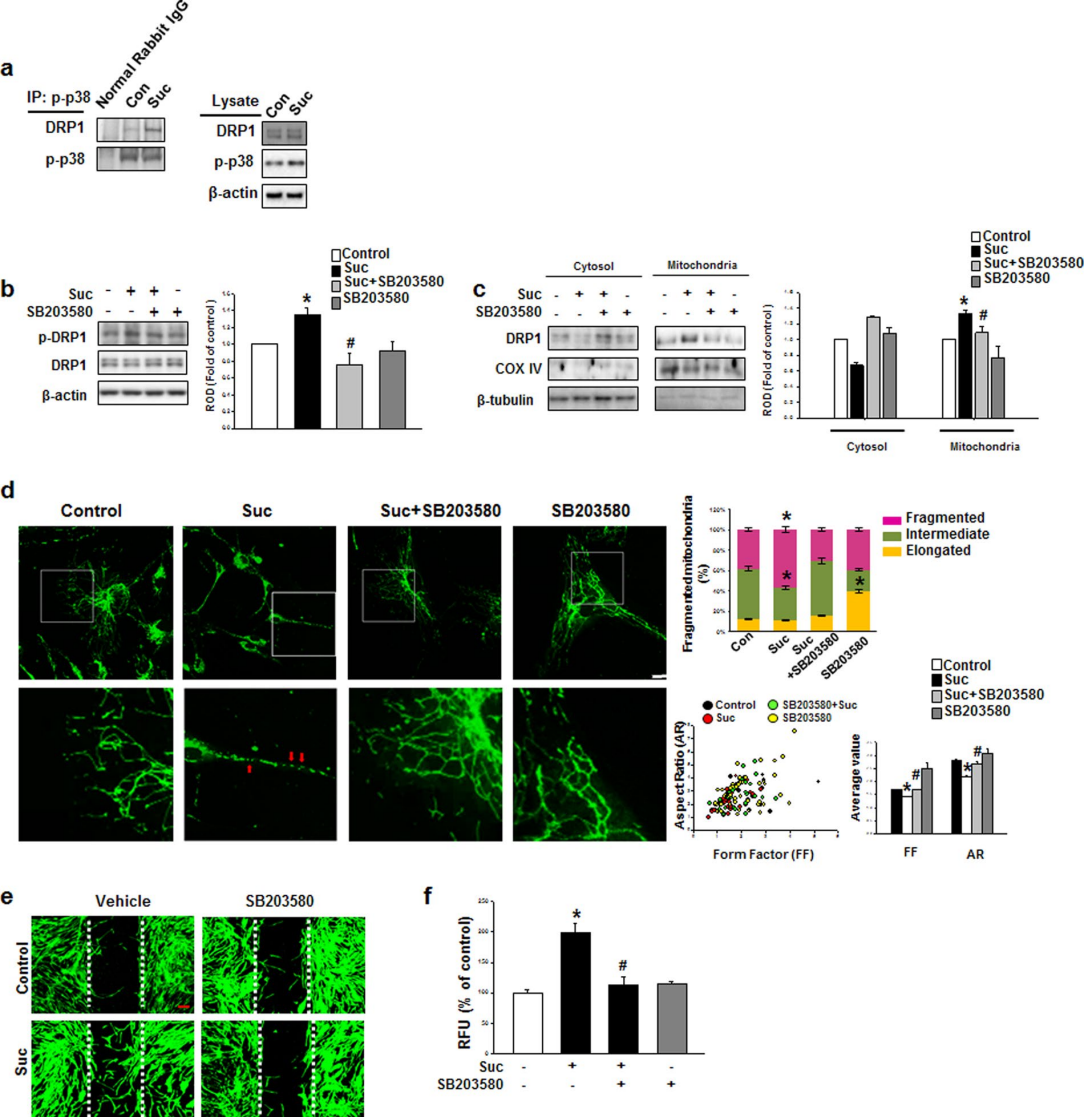
Succinate-induced p38 MAPK activation regulates DRP1 phosphorylation. **(a)** Cells were treated with succinate for 12 hr and the total lysate were used for performing immunoprecipitation using normal rabbit IgG or p-p38 antibody to determine the protein binding. **(b)** hMSCs were with/without SB203580 (1 μM) for 30 min, and then exposed to succinate for 12 hr. The cell lysates were analyzed with western blotting, and detected with phospho-DRP1 antibody. Data denote the mean ± SEM. n = 3, ^*^p < *0*.*05* versus control, ^*#*^*p* < *0*.*05* versus succinate alone. **(c)** Cells were pretreated with SB203580 (1 μM) for 30 min before succinate treatment for 12 hr and lysate was performed for mitochondrial fractionation. Data represent the mean ± SEM. n = 3, ^*^p < *0*.*05* versus control, ^*#*^*p* < *0*.*05* versus succinate alone. **(d)** hMSCs were pre-treated with SB203580 (1 μM) for 30 min and then exposed to succinate and then loaded with MitoTracker Green (200 nM) for 30 min. Representative images were obtained by confocal microscopy. The individual mitochondrial length was assessed, classified into three different categories and quantified as percentage. Data denote the mean ± SEM. n = 10. Scale bar = 50 μm, magnification; ×200, ^*^p < *0*.*05* versus control. **(e)** Cells were pretreated with SB203580 (1 μM) for 30 min prior to succinate exposure for 24 hr. After incubation, wound healing assay performed with phalloidin staining to identified migrating cell. Scale bar = 50 μm, magnification; ×80. **(f)** Migrated cells were quantified with Oris migration assay. Data represent the mean ± SEM. n = 3, ^*^p < *0*.*05* versus control, ^*#*^*p* < *0*.*05* versus succinate alone. Abbreviations: RFU, relative fluorescence units; ROD, relative optical density.

We further investigated how succinate regulates hMSC mitochondrial dynamics. It has been reported that mitogen-activated protein kinase (MAPK) directly phosphorylates DRP1 which consequently lead to mitochondria fragmentation^26^. To this end, it was logical to hypothesize that succinate-induced MAPK could be the upstream-regulator of DRP1 phosphorylation. We demonstrated the succinate promoted a binding interaction between DRP1 and phospho-p38 compared to control (Fig. 4a), demonstrating that among the MAPKs, p38 MAPK is the upstream regulator of DRP1 phosphorylation. Consistently, SB203580 (p38 inhibitor) blocked DRP1 phosphorylation at Ser 616 (Fig. 4b). The translocation of DRP1 from cytosol to mitochondria also decreased by SB203580 after succinate pretreatment (Fig. 4c). To determine whether mitochondrial dynamics is involved in succinate-induced hMSC migration, mitochondria were stained with MitoTracker Green and were observed under confocal microscopy. Mitochondrial length was divided into three categories: >3 μm, 2 to 3 μm, and <2 μm. After succinate treatment, mitochondria were observed significantly fragmented compared to the control, and SB203580 treatment changed mitochondria morphology from fragmented to intermediate or elongated shape. Furthermore, the effects of succinate on mitochondrial morphology were analyzed with the AR and FF. After 24 hr succinate treatment, both AR and FF values were decreased with succinate treatment and the values were back to control level after SB203580 treatment (Fig. 4d). We observed that SB203580 significantly blocked succinate-induced hMSC migration in Ibidi wound healing assay and Oris migration assay to confirm that p38 MAPK-induced DRP1 phosphorylation was related to hMSC migration (Fig. 4e and f). These data provide an important evidence that succinate-induced p38 MAPK activation directly regulates DRP1 phosphorylation, and consequently result in mitochondrial fission which is vital to hMSC migration.

### The role of DRP1-mediated mitochondrial fission induced mitochondrial ATP production in hMSC migration

**Figure 5:**
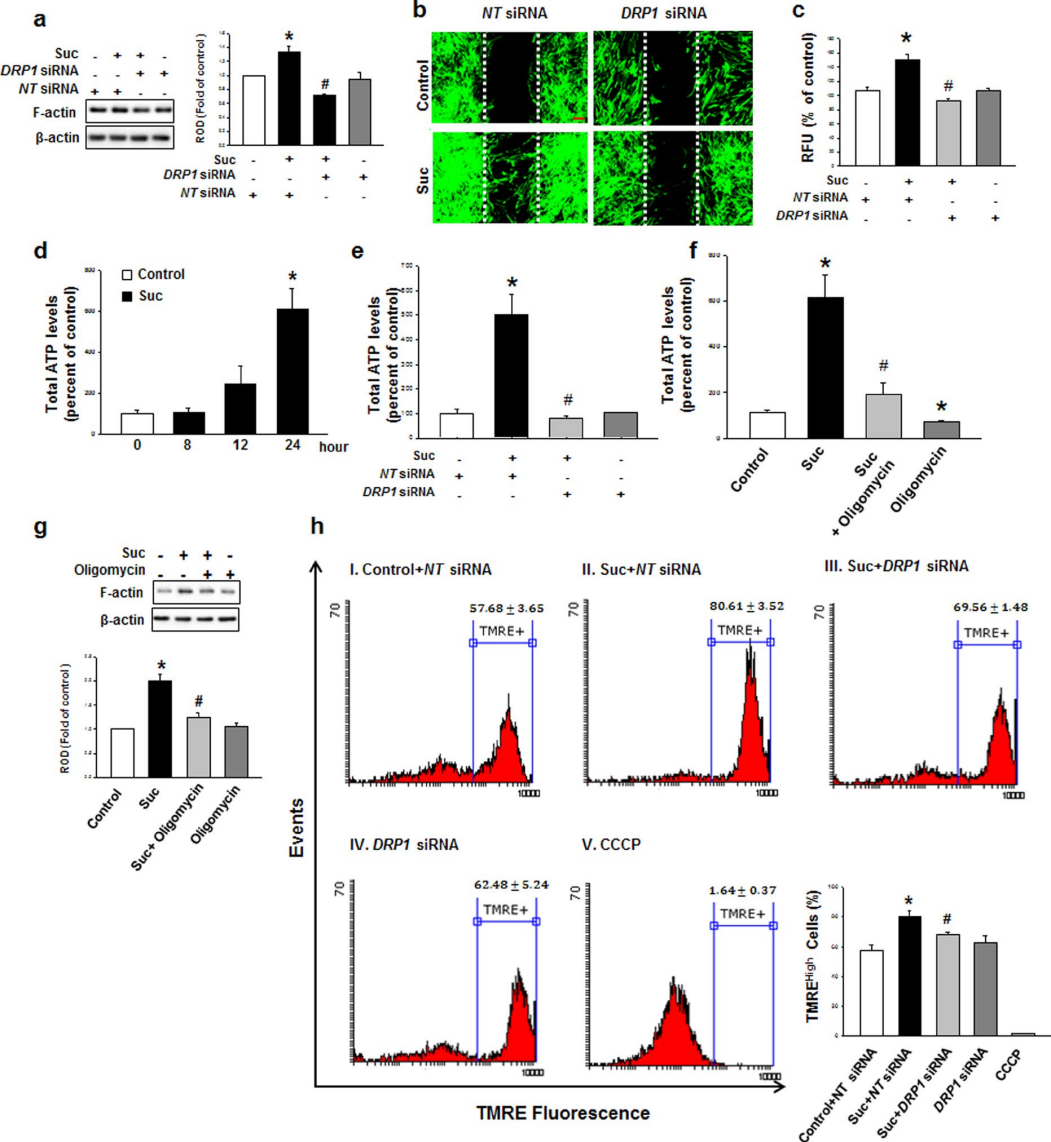
DRP1-mediated mitochondrial fission enhances mitochondrial ATP production. **(a)** *DRP1* siRNA was transfected in hMSC before succinate treatment. After incubation with succinate, cells were analyzed by western blotting with anti-F-actin antibody. Data represent the mean ± SEM. n = 6, ^*^p < *0*.*05* versus *NT* siRNA, ^*#*^*p* < *0*.*05* versus succinate with *NT* siRNA. **(b)** Cells were cultured in ibidi confocal dish and transfected with *DRP1* siRNA. Then cells were stained with phalloidin and observed with confocal microscopy. Scale bar = 50 μm, magnification; ×80. **(c)** Migrated cells were quantified with Oris^™^ migration assay. Data represent the mean ± SEM. n = 3, ^*^p < *0*.*05* versus control, ^*#*^*p* < *0*.*05* versus succinate with *NT* siRNA. **(d)** After incubation of hMSC with/without succinate, cells were lysed and reacted with ATP luciferase reagent. Then ATP levels were detected with luminometer. Data represent the mean ± SEM. n = 6. ^*^p < *0*.*05* versus control. **(e)** Cells were transfected with *DRP1* siRNA prior to succinate treatment. Total cellular ATP levels were analyzed using ATP assay kit to detect luminescent ATP. Data represent the mean ± SEM. n = 6, ^*^p < *0*.*05* versus *NT* siRNA, ^*#*^*p* < *0*.*05* versus succinate with *NT* siRNA. **(f)** hMSCs were pretreated with oligomycin before succinate treatment. Total ATP levels were represented mean ± SEM. n = 6. ^*^p < *0*.*05* versus control, ^*#*^*p* < *0*.*05* versus succinate. **(g)** hMSCs were treated with Oligomycin (1 μM) for 1 hr prior to expose to succinate. Cells were harvested subjected to western blot and detected using F-actin antibody. Data represent the mean ± SEM. n = 3, ^*^p < *0*.*05* versus control, ^*#*^*p* < *0*.*05* versus succinate. **(h)** hMSCs were treated with succinate for the indicated time, and mitochondrial memebrane potential was asscessed by TMRE staining. Mitochondrial membrane potential stained with TMRE (400 nM) was measured using FACS analysis (Total cell counts = 5 × 10^3^ cells). CCCP (20 μM) treatment for 1 hr was used for negative control. Data represent the mean ± SEM. n = 3, *p < *0*.*05* versus control + *NT* siRNA, ^*#*^*p* < *0*.*05* versus succinate + *NT* siRNA. Abbreviations: ROD, relative optical density.

To elucidate the role of DRP1 in the succinate-induced hMSC cytoskeleton reorganization, *DRP1* siRNA was transfected into hMSC. Succinate-induced hMSC F-actin formation was decreased to the control level (Fig. 5a) and hMSC migration was attenuated (Fig. 5b and c) after *DRP1* siRNA transfection. We subsequently determined whether mitochondrial fission by succinate treatment affects the mitochondrial functions. After 24 hr succinate treatment, total ATP levels were increased (612%) compared to the control (Fig. 5d). We confirmed that *DRP1* siRNA transfection attenuated succinate-induced ATP levels to the control level (Fig. 5e). To determine whether succinate-induced ATP was related to mitochondrial ATP, we treated Oligomycin, which inhibits mitochondrial F1F0-ATPase. Oligomycin downregulated succinate-induced ATP levels to the control level (Fig. 5f), confirming that succinate-induced hMSC ATP enhancement is associated with mitochondrial ATP production^31^ . Additionally, Oligomycin treatment attenuated succinate-induced F-actin formation (Fig. 5g). We also examined the mitochondrial membrane potential (ΔΨm) level through TMRE staining^32^, for mitochondrial respiration occurs reciprocally with electrochemical proton gradient^33^. Succinate also increased ΔΨm indicating the enhancement of mitochondrial energy production, and this was attenuated after cells were transfected with *DRP1* siRNA (Fig. 5h). Taken together, our results revealed succinate-induced DRP1-mediated mitochondrial fission increased mitochondrial membrane potential.

### Succinate-induced mtROS enhanced F-actin reorganization through Rho GTPase Activation

**Figure 6:**
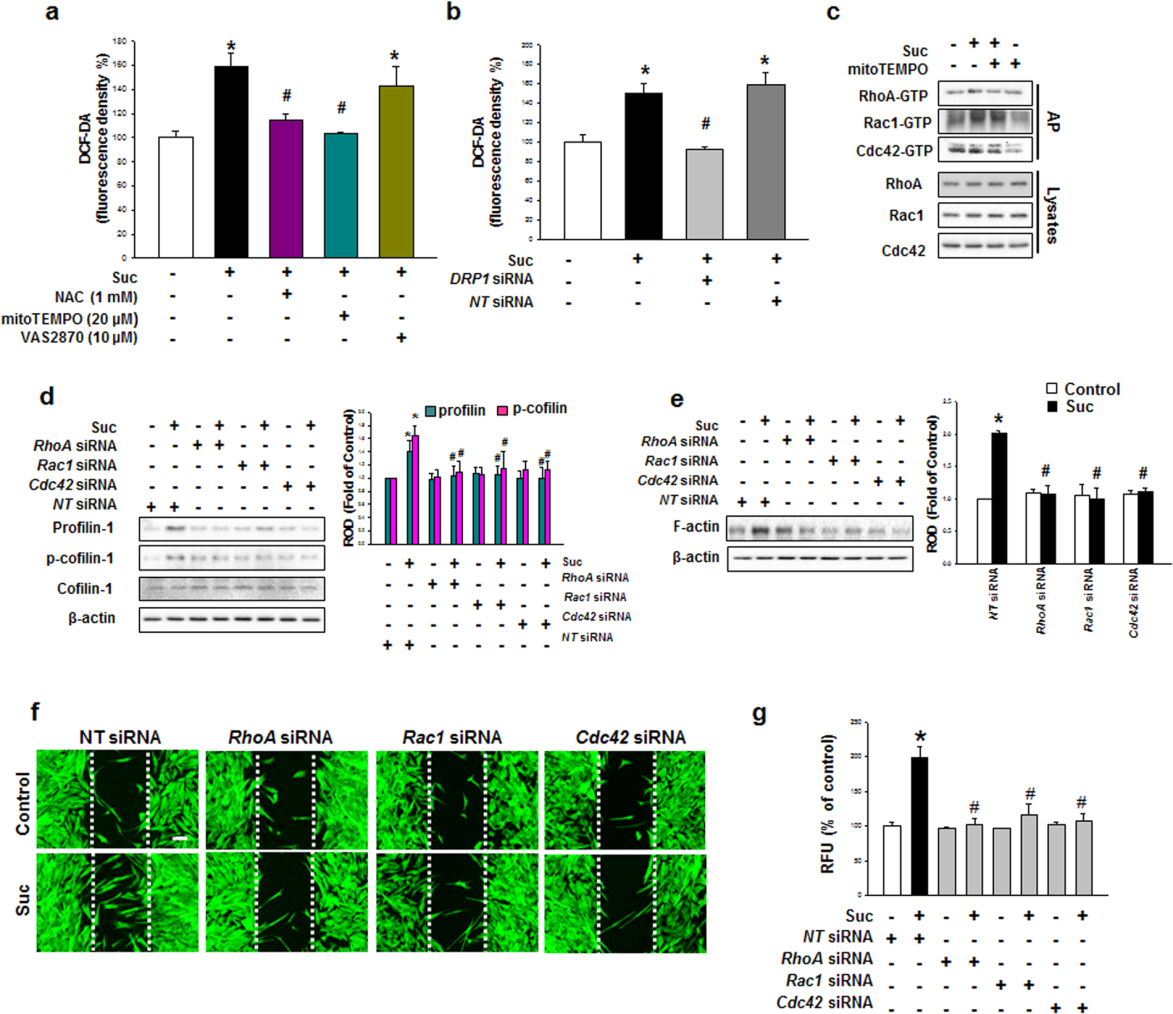
Involvements of mtROS in F-actin formation. **(a)** hMSC was pretreated with NAC, mitoTEMPO and VAS2870 in and incubated with succinate. Then hMSC was stained with DCF-DA and ROS levels were measured using luminometer. Data represent the mean ± SEM. n = 3, ^*^p < *0*.*01* versus control, ^*#*^*p* < *0*.*05* versus succinate. **(b)** hMSC was transfected with *DRP1* siRNA and treated with succinate for 24 hr. Data represent the mean ± SEM. n = 6, ^*^p < *0*.*05* versus control, ^*#*^*p* < *0*.*05* versus succinate. **(c)** hMSC was treated with mitoTEMPO (20 μM) prior to expose to 12 h succinate and the total lysates were incubated with agarose bead. The bound activated GTP-RhoA, GTP-Rac1, GTP-cdc42 were dectected with western blot analysis. **(d)** Cells were transfected *RHO GTPases* siRNA and then treated with succinate. Western blot performed to detect profilin, p-cofilin and cofilin protein expression. Data represent the mean ± SEM. n = 3, ^*^p < *0*.*05* versus control with *NT* siRNA, ^*#*^*p* < *0*.*05* versus succinate with *NT* siRNA. **(e)** *RHO GTPases* siRNA transfected cells were treated for 24 hr and F-actin protein expression was detected with western blotting. Data represent the mean ± SEM. n = 3, ^*^p < *0*.*05* versus *NT* siRNA alone, ^*#*^*p* < *0*.*05* versus succinate with *NT* siRNA. **(f)** hMSC was cultured with ibidi dish and transfected with *RhoA*, *Rac1*, and *Cdc42* siRNA prior to 24 hr succinate treatment. Cells were stained with phalloidin and observed with confocal microscopy. **(g)** *RHO GTPases* siRNA-transfected cell was incubated with succinate and the hMSC migration was quantified with Oris^™^ migration assay. Data represent the mean ± SEM. n = 4, ^*^p < *0*.*05* versus *NT* siRNA alone, ^*#*^*p* < *0*.*05* versus succinate with *NT* siRNA. Abbreviations: ROD, relative optical density.

Increased mitochondrial respiration induces ROS production in various cells. Therefore, we confirmed succinate-induced ROS production using DCF-DA to detect the total cellular ROS level. After succinate treatment, ROS production was increased after 1 hr in a time-dependent manner (Supplementary Fig. S2a and b). Generally, ROS is made from mitochondria and NADPH oxidase which can be inhibited by mitoTEMPO and VAS2870 respectively, while N-acetylcysteine (NAC) can attenuate all two sources of ROS^34^. We treated mitoTEMPO (20 μM), VAS2870 (10 μM) and N-acetylcysteine (NAC, 1 mM) for 30 min prior to the exposure to succinate for 12 h. Our data demonstrated that succinate-induced ROS was inhibited by NAC and mitoTEMPO, indicating that the mitochondria were the major source of succinate-induced ROS production (Fig. 6a). Moreover, *DRP1* siRNA transfection attenuated mtROS, indicating that ROS was generated during DRP1-mediated mitochondrial fission (Fig. 6b). ROS is widely known to enhance cytoskeleton reorganization, and accordingly, we investigated the effect of mitochondrial ROS (mtROS) on the cytoskeletal dynamic-related small GTPases. Affinity precipitation for Rho GTPases revealed that the activities of RhoA, Rac1 and Cdc42 were increased by succinate treatment in a time dependent manner (Supplementary Fig. S3). To determine whether mtROS had an effect on Rho GTPases, cells were treated with mitoTEMPO, which resulted in the downregulation of RhoA, Rac1 and Cdc42 (Fig. 6c). Consistently, *RhoA*, *Rac1* and *Cdc42* siRNA significantly blocked the succinate-induced p-cofilin and profilin activation, which are responsible for cell motility. (Fig. 6d). *RhoA GTPase* siRNA transfection significantly impaired F-actin protein expression (Fig. 6e). In addition, *RhoA*, *Rac1* and *Cdc42* siRNA transfection consequently suppressed hMSC migration to denuded area (Fig. 6f) and as demonstrated in Oris migration assay (Fig. 6g). These results demonstrated that the increase of mtROS consequently enhances hMSC migration through Rho GTPase activation.

### Succinate-Induced DRP1 Mediated Mitochondrial Fission Involved in Skin Regeneration Processes

**Figure 7:**
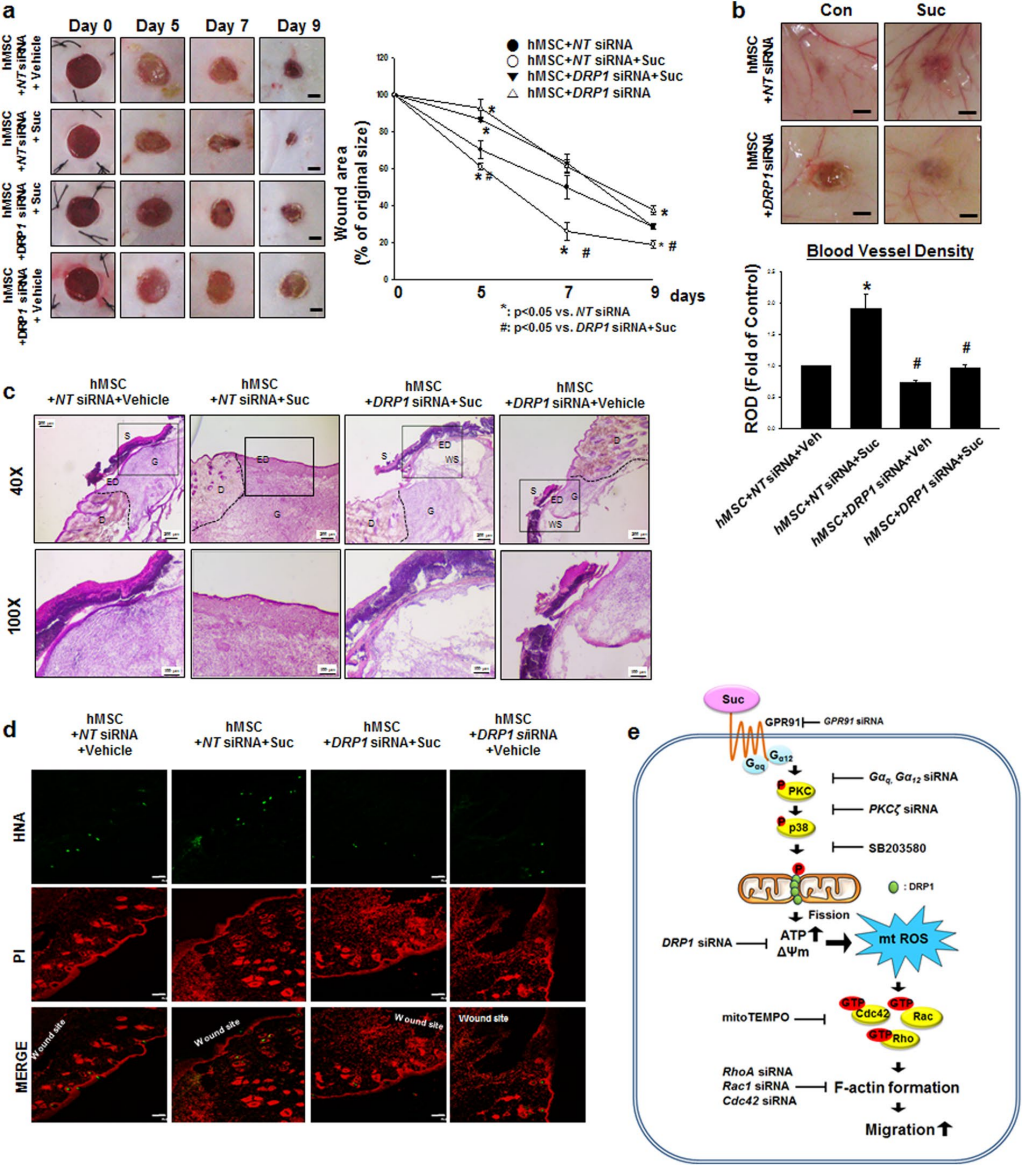
The role of DRP1 on skin wound healing *in vivo*. **(a)** Representative gross images of skin wound healing at day 0, 5, 7, and 9 are shown. Mouse skin wounds were surgically made by 6-mm-diameter biopsy punch and treated with hMSC + *NT* siRNA + vehicle, hMSC + *NT* siRNA + succinate, hMSC + *DRP1* siRNA + succinate or hMSC + *DRP1* siRNA + vehicle, respectively. ***(left panel)***. Wound healing was determined by assessing the percentage of wound closures relative to original wound size. Data represent mean ± SEM. n = 6, ^*^p < *0*.*05* versus *NT* siRNA, ^*#*^*p* < *0*.*05* versus *DRP1* siRNA + succinate ***(right panel****)*, Scale bars = 2 mm. **(b)** The quantified vascularity wound area at day 9 was shown. Data represent mean ± SEM. n = 6, ^*^p < *0*.*05* versus hMSC with *NT* siRNA, ^*#*^*p* < *0*.*05* versus hMSC with *NT* siRNA and succinate, Scale bars = 2 mm. **(c)** Representative wound tissues stained with H&E at day 9 were shown. n = 6, Scale bars = 200 μm or 100 μm, magnification; ×40 or ×100, respectively. **(d)** Engraftment of hMSCs on wound at day 9 was observed by using confocal microscopy. Human nuclear antigen (HNA, green) was used for hMSC nuclear staining. Propidium iodide (PI, red) was used for nuclear counter staining, Scale bars = 100 μm, magnification; ×100. **(e)** Hypothetical model for the effect of succinate on hMSC migration through mitochondrial fission. Succinate induced PKC phosphorylation through GPR91 activation. Then PKC activated p38 MAPKs and subsequently DRP1 phosphorylation. DRP1 phosphorylation caused mitochondrial fission and finally enhancement of mitochondrial ATP production and mitochondrial membrane potential. In result of increased mitochondrial functions, mtROS was produced and induced Rho GTPases activation. Finally, activated Rho GTPases caused F-actin formation to promote hMSC motility. Abbreviations: S, scab; D, dermis; ED, epidermis; G, granular tissue; WS, wound site; ΔΨm, mitochondrial membrane potential.

Before confirming the role of DRP1 in skin wound healing, we explored the effect of hMSC pre-treated with succinate (50 μM) on skin wound healing in mice. Consequently, we found mice transplanted with succinate pretreated hMSC showed faster wound restoration compared to the other group (Supplementary Fig. S4). To confirm the role of DRP1 on succinate-induced stem cell migration, hMSC was transfected with *DRP1* siRNA or *NT* siRNA. As a result, the wound size (%) in the succinate pretreatment with hMSC group were considerably smaller than in the *NT* siRNA + vehicle treated hMSC group on days 5 and 7. To verify the role of succinate during hMSC migration reciprocally DRP1-mediated mitochondrial fission, *DRP1* siRNA was transfected into hMSCs, which showed considerably delayed cutaneous wound curing compared to the vehicle (Fig. 7a). Simultaneously, the effect of succinate pretreatment on the degree of vascularization was significantly attenuated in *DRP1* siRNA transfected hMSCs, as observed in thin and unmatured blood vessel branches (Fig. 7b). A histological examination at day 9 showed that the wound healing was extremely delayed in succinate + *DRP1* siRNA treated hMSC group despite the succinate treatment, whereas hMSC + *NT* siRNA + succinate treatment led to almost complete recovery with well-organized granulation tissue and complete remodeling of the thick cornified epidermis (Fig. 7c). Concurrently, hMSC + succinate + *NT* siRNA treated mice group showed significantly increased number of cells that migrated to the wound edge that gradually covered the wound surface. However, the mice group treated with hMSC with *DRP1* siRNA transfection showed failed stem cell transplantation with a failure to migrate to the wound site (Fig. 7d). These results indicates that ex-vivo stimulation of hMSC with succinate promotes the injured tissue regeneration through the functional enhancement of DRP1-mediated hMSC.

## Discussion

In this study, we clearly demonstrated that succinate promotes hMSC migration through PKCζ/p38-induced DRP1 phosphorylation which upregulates mtROS production subsequent to F-actin formation, collectively accelerating the wound repair. Succinate is a charged molecule, therefore it binds to its specific receptor, GPR91, under a physiological condition^10^. The physiological response to succinate varies depending on its concentration. In previous reports, a high succinate concentration (10 mM) with prolonged incubation induced cell apoptosis, whereas succinate concentration at the physiological level played an essential role in hepatic stellate cell regeneration through the GPR91 signaling pathway, indicating that ex-vivo hMSC stimulation with succinate can be a target to promote the wound healing processes^35,36^. We confirmed the importance of an adequate succinate concentration (50 μM) and time (24 h) on the enhancement of hMSC motility at physiological level. GPR91 interacts with various Gα proteins, including Gα_i_ or Gα_q_, and triggers diverse pathways depending on the specific cell type^37^. However, in our study, Gα_q_, Gα_i,_ Gα_12_ are activated by succinate, of which Gα_q_ and Gα_12_ were specifically involved in hMSC migration. GPR91 becomes activated by either Gα_i_ or Gα_q_ through Ca^2+^ upregulation, which consequently stimulates PLC to activate PKC^10,38^. An earlier study showed that Gα_q_ and Gα_12_ double-knock out mouse embryos died in utero, although an individually deficient mouse survived, suggesting that Gα_q_ and Gα_12_ play pivotal roles in cell functions via their interactive actions^39,40^. Moreover, it has been reported that protease-activated receptor 1 activation by thrombin is involved in both Gα_q_ and Gα_12_ which is correlate with PKC-related kinase stimulation^41^. In this regard, we detected PKC phosphorylation and our study showed that succinate did not alter Ca^2+^ mobilization but activated Ca^2+^-independent atypical types of PKCζ. We then focused on the downstream effector of PKCζ. It has been reported that stromal cell-derived factor-1 induces hMSCs migration through MAPK activation, indicating that MAPK can be a targeted downstream effector of PKCζ^42^. Moreover, GPR91 activation induced the vascular endothelial growth factor release by mediating p38 MAPKs signaling in retinal ganglion cells^43^. Consistent with those results, our data showed that *PKCζ *siRNA transfection significantly decreased p38 MAPK phosphorylation. Thus, all the results indicate that succinate induces p38 MAPK phosphorylation through Gα_q_ and Gα_12_ dependent PKC activation.

It has been reported that mitochondrial dynamics can tightly regulate cellular bioenergetic roles and homeostasis. This prompted us to investigate the relevance of succinate and mitochondrial dynamics on hMSC migration^19,20^. Therefore it is necessary to investigate the mitochondrial dynamics protein expression level and post-translational modifications to elucidate the signaling pathways that control DRP1 activities^44^. Our study demonstrated that there were no significant alterations in the mitochondrial dynamics-related protein mRNA expression levels, except for the phosphorylation of DRP1 at the Ser 616 site (Supplementary Fig. S5a and b). Abounding evidences showed that DRP1 is phosphorylated by various upstream regulators including CDK1, CDK5, and Akt in different cell functions depending on specific cell types^26,45,46^. In this regard, we treated mitomycin C prior to the exposure to succinate, for most upstream regulator of DRP1 phosphorylation involves in cell proliferation. However, succinate did not affect hMSC proliferation but promoted migration. We therefore focused on upstream regulators of mitochondrial fission that affect hMSC migration. Our results clearly indicated that p38 MAPK directly binds to DRP1 after succinate treatment. Our findings are further supported by a previous study reporting that DRP1 phosphorylated by ERK MAPK signaling pathway leads to increased mitochondrial fission and consequently cell reprogramming^26^. In addition, phosphorylation of DRP1 leads to mitochondrial fission, according to our fragmented morphology analysis with mitotracker staining. Mitochondrial fission can be a double-edged sword depending on the cellular condition. Mitochondrial fission occurs in a pathophysiological condition, resulting in depletion of energy production, mitochondrial depolarization and finally mitophagic degradation^46,47^. On the other hand, in a physiological condition, fragmented mitochondria are redistributed equally in proliferative cells, enhancing mitochondrial respiration which are essential in maintaining cell polarity^22,23,45^. Although several studies have shown that mitochondrial fission protein DRP1 plays a role in cell migration by increasing the mitochondrial respiration, the detailed molecular mechanism remains poorly understood^24,25^. The localization of mitochondria is important for appropriate supplying ATP and regulating Ca^2+^ concentration in the neuron^23^. The fragmented mitochondria may be transported to place with energy demands. In this regards, we showed that succinate-induced fragmented mitochondria redistributed along the F-actin generation (Supplementary Fig. S6). Also we showed that DRP1-mediated mitochondrial fission plays an important role in hMSC migration by enhancing ATP production with ΔΨm. Our data is supported by a report that mitochondrial fission generates two uneven daughter organelles, consequently making one with high membrane potential and the other with low membrane potential, suggesting that mitochondria division could contribute to mitochondrial quality control by increasing membrane potential which is crucial in bioenergetic demands^48^. This is the first finding to report that succinate-mediated mitochondrial fission regulated the functional mitochondrial activities of hMSC which resulted in cell migration. Our study clearly revelaed that DRP1-mediated mitochondrial fragmentation by succinate is vital in enhancing mitochondria functions, indicating that succinate-induced mitochondrial morphology alteration can regulate hMSC physiology.

Interestingly, our results showed that succinate-induced mitochondrial fission enhanced mitochondrial energy production suggesting that by-product of cellular respiration, mtROS, would be a mediator of succinate-induced hMSC migration. Our results are further supported by the report that high glucose level leads to mitochondria fragmentation, which is necessary to increase mitochondrial function, consequently inducing ROS production^49^. To this end, we hypothesized that succinate-induced mtROS can be a mediator in initiating cell signaling for migration. An earlier study has shown not only that mtROS occupies largely intracellular ROS production but also that the time and quantity of ROS is important in cell homeostasis through coordinating regulation of mitochondrial morphology^50,51^. Succinate treatment induced the sustained mtROS production and there is compelling evidence that sustained ROS is involved in the wound healing process, indicating that succinate-induced sustained ROS has a functional role in the area of stem cell physiology^51,52^. There are also several reports which strongly support our study that mtROS can induce cell migration via various forms of downstream signaling in endothelial and tumor cells^53^. However, the role of mtROS in stem cell has yet to be elucidated, and to date, this is the first study to suggest that mtROS functions as a signaling molecule in promoting hMSCs migration. ROS activates the Rho small GTPase family in relation to cofilin, profilin activity which the major regulator of actin polymerization^54^. Cell migration is tightly regulated by the interaction of RhoA, Rac1 and Cdc42, depending on the specific cell types. In bone marrow derived hMSC, only Rac1 and Cdc42 induce cell migration while RhoA is involved in cell contraction^55,56^. Also LPA-induced umbilical-cord blood derived hMSC migration was increased by interplaying only between Rac1 and Cdc42, indicating that cell migration could be regulated by specific ligands initiating different signaling pathway even within the same cells^57^. Although it has been reported that activated RhoA can lead to the inactivation of Rac1, we showed RhoA, Rac1 and Cdc42 reciprocally regulates cytoskeletal reorganization, suggesting that mtROS-mediated Rho effector ROCK signaling pathway can regulate cell migration in wound healing processes^51^. This study is further supported by the reports that Rho small GTPase family coordinately acted in actin cytoskeletal rearrangements, which is crucial in hematopoietic stem cells^55^. Correspondingly, Rho could act positively or negatively on stem cell migration in accordance with its spatial expression levels and specific cell type^57^. Collectively, it is not clear whether succinate influences additional cytoskeleton reorganization such as loss of cell adhesion or other independent cellular signaling activities, but our data showed that succinate promotes hMSC migration as a consequence of RhoA, Rac1 and Cdc42 activation which subsequently regulates profilin and cofilin activity.

We showed *in vivo* evidences that the transplantation of succinate-prestimulated hMSC accelerates skin wound healing. Succinate has been observed to enhance angiogenesis, suggesting the possibility of succinate to accelerate injured tissue regeneration^14,58^. Contrary to previous reports, succinate alone treated mice group showed no significant effect on accelerating vascularity, suggesting that succinate might have therapeutic potential only when treated with hMSCs. In wound healing processes, hMSC migrates to the wound site in response to chemotaxis signaling, which promote angiogenesis and induces cell migration^59^. Furthermore, our *in vitro* data clearly showed that succinate-induced mitochondrial fission regulated hMSC motility. For this reason, the role of DRP1-mediated mitochondrial fission in actual wound healing processes need to be further investigated for more useful stem cell therapy. Our study demonstrated that *DRP1* siRNA transfection significantly delayed tissue regeneration and blood vessel regeneration, implying that DRP1-mediated mitochondrial fission has the ability to increase hMSC function in the restoration of damaged tissues. Therefore, our *in vivo* data confirms that succinate is a very novel bioactive molecule in modulating mechanistic functions of hMSC that regulates stem cell mitochondrial function in regenerative medicine with an economical and timesaving aspect. For these reasons, understanding and manipulating metabolic regulations of hMSCs can provide a new strategy in modulating stem cell behavior for safe and beneficial clinical applications. In conclusion, succinate induced PKCζ/p38 MAPK activation directly causes DRP1-mediated mitochondrial fission to increase mitochondrial function. mtROS generation activated Rho GTPases and F-actin assembly to promote hMSC motility which consequently improves the therapeutic efficacy of hMSC in wound healing *in vivo* (Fig. 7e).

## Materials and Methods

### Materials

hMSCs were provided from the Medipost, Co. (Seoul, Korea). Fetal bovine serum was purchased from Bio Whittacker (Walkersville, MD, USA). The following antibodies were purchased: F-actin, phospho-PKC, phospho-DRP1, Gα_q_, Gα_i_ and Gα_12_ antibodies (Cell Signaling Technology, Danvers, MA, USA); Cdc42, Rac1 and RhoA antibodies (BD Biosciences, Franklin Lakes, NJ, USA); DRP1, OPA1, Fis1, MFN2, p-ERK, p-JNK, ERK, JNK, GPR91, PKCδ, PKCε, PKCθ, PKCζ, profilin-1, phospho-cofilin1, cofilin1, β-actin and and β-tubulin antibodies (Santa Cruz Biotechnology, Paso Robles, CA, USC); COX IV antibody (Abcam, Cambridge, England); MFN2 antibody (Proteintech, Wuhan, China); Horseradish peroxidase (HRP)-conjugated goat anti-rabbit IgG (Jackson Immunoresearch, West Grove, PA, USA). Succinate, NAC and mitomycin C were obtained from Sigma Chemical Company (St. Louis, MO, USA).

### Cell culture

hMSCs were cultured in α-minimum essential medium (Thermo, MA, USA) with 1% antibiotics (penicillin/streptomycin) and 10% FBS. Cells were grown at 37 °C with 5% CO_2_ in the incubator. When cells were grown 70% confluence, the medium was replaced with serum-free medium excluding all supplements at 24 hr before experiments.

### Mouse excisional wound splinting model

Eight-week-old male ICR mice were used. All animal procedures were performed with following the National Institutes of Health Guidelines for the Humane Treatment of Animals, with approval from the Institutional Animal Care and Use Committee of Seoul National University. Mice were anesthetized deeply with 3% isoflurane in a mixture of N_2_O/O_2_ gas^60,61^. To test the functional role of hMSCs pretreated with succinate, the mice were randomly divided into four groups: wild-type mice that were treated with vehicle (group 1, n = 6) or 50 μM succinate (group 2, n = 6); hMSCs skin implantation group mice that were given succinate-stimulated hMSC (group 3, n = 6) or that were given only hMSC (group 4, n = 6). To determine the role of DRP1 in migration of hMSC to wound area for cutaneous regeneration, the mice were grouped into hMSCs transfected with *DRP1* siRNA with (group 5, n = 6)/without (group 6, n = 6) succinate stimulation or *Non-targeting* (*NT*) siRNA with (group 7, n = 6)/without (group 8, n = 6) succinate pretreatment. The back was shaved, sterilized with povidone iodine followed by 70% ethanol. The wound was created with using a 6 mm diameter sterile biopsy punch surgically. The hMSCs were pretreated with 50 μM succinate for 24 hr before the skin implantation. In the cell-treated group, 1 × 10^6^ hMSC in 100 μl saline were injected into the dermis at three sites around the wound. After the cell transplantation, splints were placed around the wounds (with glue and several stitches) and then wounds were dressed with Tegaderm (3 M, London, ON, Canada) sterile transparent dressing. Mice were placed in individual cages in a clean facility. Images of wound were acquired on day of 0, 5, 7 and 9 with a digital camera system (40D, Canon, Tokyo, Japan) at the same distance (30 cm). At day 9, the wound tissues were embedded in OCT compound (Sakura, Finetek, Torrance, CA, USA) stored at −70 °C, cut 6-μm-thick frozen sections using cryosectioning machine and mounted on SuperFrost Plus slides (Thermo Fisher Scientific, Waltham, MA, USA) for haematoxylin and eosin (H&E) staining and immunohistochemistry.

### Small interfering (si) RNA transfection

Cells were grown in 70% confluence and transfected for 24 hr with *DRP1*, *Gα*_q_, *Gα*_i_, *Gα*_12_, *RhoA*, *Rac1*, *Cdc42* and *PKCζ* siRNA (25 nM; Dharmacon, Lafayette, CO, USA), or *NT* siRNA (25 nM; Dharmacon, Lafayette, CO, USA) as a negative control using Turbofect Transfection reagents (Thermo Fisher Scientific, Waltham, MA, USA) according to the manufacturer’s instructions.

### Wound-healing migration assay

Cells were seeded at 4 × 10^3^/μl in the both silicone reservoirs of 35-mm dishes (Ibidi, Martinsried, Germany). When cells were grown around 90% confluence, the medium was replaced with serum-free medium. After 24 hr incubation, the silicone reservoirs were carefully removed with sterile forceps for allowing the wound field. The cells were incubated another 24 hr with succinate and/or siRNA transfection and were incubated with phalloidin. Then the cells stained with phalloidin were visualized with an Olympus FluoView (Olympus, center Valley, PA, USA) 300 confocal microscope with 80× objective.

### Oris cell migration assay

Cells were seeded at 3 × 10^3^ cells/100 μL in Oris well (Platypus Technologies, Fitchburg, WI, USA) and were incubated for 24 hr to permit cell adhesion. When cells were grown 80% confluence, inserts were carefully removed, then cells were incubated with succinate (50 μM) and serum-free medium. After incubation, cells were stained with 5 μM calcein AM for 30 minutes and migrated cells were quantified by fluorescence signals measurement using Victor3 luminometer (PerkinElmer, Inc., Waltham, MA, USA) at excitation and emission wavelengths of 485 nm and 515 nm.

### Reverse transcription polymerase chain reaction and real-time PCR

The hMSC RNA was extracted with the RNeasy Plus Mini Kit (QIAGEN, Valencia, CA, USA). Reverse transcription was performed with 3 μg of RNA using a Maxime RT premix kit (iNtRON biotechnology, Seongnam, South Korea). Real-time quantification of RNA targets was performed with Rotor-Gene 6000 thermal cycling system (Corbett Research, NSW, Australia) using QuantiTect SYBR green PCR kits (QIAGEN, Hilden, Germany). The reaction mixture (20 μl) contained 200 ng of RT products, 0.05 μM of each primer, and appropriate amounts of enzymes and fluorescent dyes according to a manufacturer’s instructions (Supplementary Table S1). The fluorescent intensity was acquired during the extension step and melting curve analysis was performed to compare the expression of RNA targets.

### Western blot analysis

Harvested cells were lysed with RIPA lysis buffer for 30 min on ice. Protein concentration was determined by the Bradford method. Equal amounts of protein (20 μg) were resolved by 10% sodium dodecyl sulfate polyacrylamide gel electrophoresis (SDS-PAGE) and were transferred to polyvinylidene fluoride membranes. Membranes were incubated with indicated antibody followed by ECL treatment for detecting signals. The results were analyzed by optical density using Image J program.

### Mitochondrial morphology analysis

According to the manufacturer’s instructions, cells were cultured in confocal dish and incubated with Mitotracker Green FM (200 nM) for 30 min at 37 °C incubator. After incubation, cells were washed with pre-warmed media. Mitotracker Green-stained cells were visualized with confocal microscopy (×400). Quantitative analysis of mitochondrial morphology was performed by using FIJI software. Mitochondrial morphology analyzed after thresholding. Individual mitochondria particles analyzed for circularity (Form factor = (perimeter)^2^/(4π * area)) and lengths ratio of major and minor axes. Form factor (FF) indicates mitochondrial length and branching and Aspect ratio (AR) imply length of the mitochondria. When FF and AR both increase, the mitochondria morphology becomes branched and elongated^62^.

### Measurement of calcium influx

Fluo-3AM dissolved in dimethylsulfoxide was used to detect the intracellular Ca^2+^ levels. Cells in 35-mm confocal dish were washed with PBS solution and incubated with a bath solution including 2 mM Fluo-3AM for 1 hr. After Fluo-3AM staining, cells were washed twice with PBS solution and scanned with Olympus FluoView 300 confocal microscope with 300× objective.

### ATP assay

The intracellular ATP was measured with ENLITEN ATP assay kit (Promega, Madison, WI, USA) according to manufacturer’s instructions. Briefly, cells were boiled with lysis buffer at 100 °C for 5 min. Then the cell lysate centrifugated at 15,000 rpm for 2 min. The extraction was collected and kept on ice. The same volume of the lysate and rL/L reagent were incubated in the 96-well-plate. ATP concentration was normalized with ATP standard curve using ATP standard solution (10 μM).

### Affinity precipitation

Activated RhoA, Rac1 and Cdc42 were detected with an affinity precipitation assay kits (EMD Millipore, Billerica, MA, USA) according to manufacturer’s instructions. Lysed cells were incubated for 1 hr with agarose bead coupled with Rho-binding domain of rhotekin (GST-Rhotekin-RBD) or Rac/Cdc42-binding domain (GST-PAK-PBD). After the incubation, agarose bead coupled with the lysate were eluted with 2 × laemmli sample buffer and analyzed with western blot using RhoA, Rac1 and Cdc42 antibodies, respectively.

### Co-immunoprecipitation (IP)

Cells lysed with the IP buffer (200 μg) were mixed with 10 μg of DRP1 antibody. The cell lysates were incubated for 4 hr with the antibody and then next 24 hr with the protein A/G PLUS-agarose immunoprecipitation reagent (Pierce, Rockford, IL, USA). After incubation, the beads were washed twice with IP buffer, and the bound proteins were eluted by sample buffer for 5 min. Samples were analyzed by western blotting with DRP1 and p-p38 antibodies.

### Mitochondria membrane potential

The mitochondria membrane potential was evaluated with TMRE staining (abcam, Cambridge, U.K) according to the manufacturer’s instructions. Cells were stained with TMRE (400 nM) with 5% CO_2_ at 37 °C incubator for 20 min. CCCP (20 μM) were used as negative control. After incubation, cells were washed with PBS solution. The intensity of TMRE staining was determined using flow cytometry and analyzed with flowing Software 2 (developed by Perttu Terho, Turku, Finland).

### Statistical Analysis

Results were expressed as mean value ± standard error of the mean (SEM). All experiments were analyzed by ANOVA, and some experiments that needed to compare with more than or equal to three or more groups were examined by using Bonferroni-Dunn test. A p value of <*0*.*05* was considered statistically significant.

## Electronic supplementary material

Supplementary information
