# Author Correction: Succinate promotes stem cell migration through the GPR91-dependent regulation of DRP1-mediated mitochondrial fission

**DOI:** 10.1038/s41598-018-31586-0

**Published:** 2018-09-03

**Authors:** So Hee Ko, Gee Euhn Choi, Ji Young Oh, Hyun Jik Lee, Jun Sung Kim, Chang Woo Chae, Diana Choi, Ho Jae Han

**Affiliations:** 1 Department of Veterinary Physiology, College of Veterinary Medicine, Research Institute for Veterinary Science, and BK21 PLUS program for Creative Veterinary Research Center, Seoul National University, Seoul, 08826 South Korea; 2 Department of Agricultural Biotechnology, Animal Biotechnology Major, and Research Institute for Agriculture and Life science, Seoul National University, Seoul, 08826 South Korea; 3 Department of Biological Sciences, Mount Holyoke College, South Hadley, Massachusetts, 01075 USA

Correction to: *Scientific Reports* 10.1038/s41598-017-12692-x, published online 03 October 2017

The original version of this Article contained errors.

The Article incorrectly referred to ‘mitochondrial respiration’ instead of ‘mitochondrial membrane potential’. This is now corrected as follows. In the Results, under the subheading ‘The role of DRP1-mediated mitochondrial fission induced mitochondrial ATP production in hMSC migration’:

“Taken together, our results revealed succinate-induced DRP1-mediated mitochondrial fission increased mitochondrial respiration.”

now reads:

“Taken together, our results revealed succinate-induced DRP1-mediated mitochondrial fission increased mitochondrial membrane potential.”

and in the Discussion,

“mtROS generation from mitochondrial respiration activated Rho GTPases and F-actin assembly to promote hMSC motility which consequently improves the therapeutic efficacy of hMSC in wound healing *in vivo* (Fig. 7e).”

now reads:

“mtROS generation activated Rho GTPases and F-actin assembly to promote hMSC motility which consequently improves the therapeutic efficacy of hMSC in wound healing *in vivo* (Fig. 7e).”

Several original blots were missing from this Supplementary Information file that accompanies this Article. The following original blot images are now included: for GPR91 (IP and lysate), Gαq (lysate), Gαi (lysate) and Gα12 (lysate) in Figure 2a; for β-actin in Figure 2b and in Figure 3a; for pan-cadherin (atypical and novel PKC) and β-actin (atypical and novel PKC) in Figure 3c; for β-actin in Figure 3d; for t-ERK, t-JNK, t-p38 and β-actin in Figure 3e; for p38 and β-actin in Figure 3f; for p-p38 (IP and lysate), DRP1(lysate) and β-actin in Figure 4a; for p-DRP1 and β-actin in Figure 4b; for COX IV (cytosol and mitochondria) and β-tubulin (cytosol and mitochondria) in Figure 4c; for β-actin in Figure 5a, Figure 5g, Figure 6d and Figure 6e; and for RhoA (lysate), Rac1 (lysate) and Cdc42 (lysate) in Figure 6c. The original blot images have also been added for Supplementary Figures S1b, S3, S5b, and S7.

Additionally, the authors have now also included a new Supplementary Figure S7 which demonstrates validation data for siRNAs used in the study, Supplementary Figure S8 which demonstrates a positive control to Figure 1C of the Article, and Supplementary Figure S9 which demonstrates a positive control to Figure 6A of the Article.

The Supplementary Information file that accompanies the Article has now been replaced.

These changes do not alter the main conclusions of the Article.

