# Supplementary material for: Succinate promotes stem cell migration through the GPR91-dependent regulation of DRP1-mediated mitochondrial fission
Source: Sci Rep. 2017 Oct 3;7:12582. doi: 10.1038/s41598-017-12692-x (PMC5626702; doi:10.1038/s41598-017-12692-x)
Supplement: Supplementary file 1 — Supplementary information [file 41598_2017_12692_MOESM1_ESM.docx]

**Supplementary information**

**Succinate promotes stem cell migration through the GPR91-dependent regulation of DRP1-mediated mitochondrial fission**

**Author names**: So Hee Ko^a^, Gee Euhn Choi^a^, Ji Young Oh^b^, Hyun Jik Lee^a^ , Jun Sung Kim^a^, Chang Woo Chae^a^, Diana Choi^c^, and Ho Jae Han^a,*^

^a^Department of Veterinary Physiology, College of Veterinary Medicine, Research Institute for Veterinary Science, and BK21 PLUS program for Creative Veterinary Research Center, Seoul National University, Seoul, 08826, Korea

^b^Department of Agricultural Biotechnology, Animal Biotechnology Major, and Research Institute for Agriculture and Life science, Seoul National University, Seoul, 08826, Korea

^c^Department of Biological Sciences, Mount Holyoke College, South Hadley, Massachusetts 01075, USA

*Corresponding author: Ho Jae Han, D.V.M, Ph. D.

Department of Veterinary Physiology, College of Veterinary Medicine, Seoul National University,

Gwanak-ro 1, Gwanak-gu, Seoul, 08826, South Korea

E-mail: hjhan@snu.ac.kr; Tel: 82-2-880-1261; Fax: 82-2-885-2732

**Supplementary figure legends**

**Supplementary Figure S1.** mRNA and protein expression of succinate-induced GPR91 in hMSC.

(a) hMSCs were treated with succinate (50 μM) for 4 h and harvested for performing real-time PCR. The bars represents the mean ± SEM. n=3, * p < 0.05 versus control. (b) Succinate was treated to hMSC and the proteins were detected with Anti-GPR91. Data represent the mean ± SEM. n=3. * p < 0.05 versus control.

**Supplementary Figure S2.** Succinate-induced cellular ROS levels in a time-dependent manner.

**(a)** hMSCs were treated with succinate (50 μM) in a time dependent manner and then incubated with DCF-DA for 30 min. Data are reported as a mean ± SEM. n=3. N.S means ‘not significant’. **(b)** hMSCs were treated with succinate (50 μM) for 0-24 hr and incubated with DCF-DA for 30 min to detect ROS using luminometer. *^*^p < 0.01* versus control.

**Supplementary Figure S3.** Succinate induced Rho GTPase family activation.

Cells were treated with succinate for 0-24 hr and the total lysate were incubated with agarose bead. The bound activated GTP-RhoA, GTP-Rac1, GTP-cdc42 were dectected with western blot analysis.

**Supplementary figure S4.** Effect of succinate on skin wound healing in vivo.

**(a)** Mouse excisional wound splinting model were used to determining the effect of succinate on hMSC migration. Wounds were surgically made by 6-mm-diameter biopsy punch. Experimental animal were divided into four groups; control group (n=6), succinate group (n=6), hMSCs (1 × 10^6^ cells) group (n=6), and succinate pre-stimulated hMSCs (1 × 10^6^ cells) group (n=6). The representative images of skin wounds at days 0, 5, 7, and 9 are shown *(left panel)*. Determining wound healing by assessing the percentage of wound closures relative to original wound size. Data represent means ± S.E. n=6. ^*^ *p < 0.05* versus control, ^#^ *p < 0.05* versus hMSCs alone *(right panel)*, Scale bars=2 mm. **(b)** The representative images of vascularity wound area at day were shown. Data represent means ± S.E. n=6. ^*^ *p < 0.05* versus control, ^#^ *p < 0.05* versus hMSCs alone, Scale bars=2 mm. **(c)** Representative images of H&E tissue staining at day post wounding were shown. n=6. Scale bars=200 μm or 100 μm, magnification; ×40 or ×100 respectively. Abbreviations: Suc, succinate; hMSC, human mesenchymal stem cell; Con, control; D, dermis; ED, epidermis; G, granular tissue; WS, wound site

**Supplementary figure S5.** mRNA and mitochondrial dynamics protein expression by succinate.

**(a)** Cells were pretreatment with succinate for 12h and then RNA extraction was carried out. Mitochondrial dynamics protein levels were determined with real-time PCR. The bars represents the mean ± SEM n=4. N.S means ‘not significant’. **(b)** Succinate were treated for 0 - 24 h and cells were harvested for analyzing western blotting with anti-OPA1, MFN1/2, FIS1, p-DRP1, DRP1. The bars next to the panel indicated mean ± SEM for three experiments for each condition which is determined by densitometry relative to the loading control β-actin. *^*^ p<0.05* versus control.

**Supplementary figure S6.** Succinate induce mitochondria redistribution through F-actin formation.

Succinate were treated for 24 h and cells were immunohistochemistry with F-actin (green), DAPI (blue) and COX IV (red) antibodies and the staining was observed with confocal microscopy. Representative confocal images were shown determined by at least 10 images from three independent experiments. The intensity graph next to confocal images indicates the distribution of F-actin (green) and COX IV (red) and observed that F-actin and COX IV co-localized with a great portion of intensity after succinate treatment, which attenuated the ratio of co-localization in *DRP1* siRNA transfected hMSC^1^. At least 20 to 30 cells were analyzed from three independent experiments. Scale bar = 100μm. All distance and intensity measurements were determined with FIJI software.

**Supplementary Figure S7.** The effect of siRNAs on mRNA expressions of *GPR91, Gα_q_, Gα_i_, Gα_12_, PKCζ, DRP1, RhoA, Rac1, Cdc42 and NT*.

**(a-i)** Cells were transfected with siRNAs for 24 h. The mRNA expressions were analyzed by quantitative real-time PCR. The mRNA expression levels of target genes were normalized with *ACTB* mRNA expression level. Data are presented as a mean ± SEM. n=3.

**Supplementary Figure S8.** Effect of 10% FBS and mitomycin C on MSC proliferation.

Cells were pretreated with mitomycin C (1 μg/mL) for 90 min. The cells were then incubated in a serum free or 10% FBS condition for 24 h. Cell number was measured using a cell counting chamber. n=5. * p < 0.05 vs serum free medium-treated cells, # p < 0.05 vs 10% FBS-treated cells.

**Supplementary Figure S9.** Effect of hypoxia on ROS production in MSCs.

Cells were incubated in 0.5% hypoxia for 24 h. After hypoxia treatment, cells were stained with 1 μM of DCF-DA for 30 min then the fluorescence intensities of the DCF-DA-stained cells were measured using a luminometer (Victor3; Perkin-Elmer, Waltham, MA, USA). n=5. * p < 0.05 vs control.

**Supplementary Figure S10. Full-length western blot images in the figure 2.**

All western blot images are full-length blot images of representative blot data in the figure 2. Dash line box indicates cropped blot image in the figure 2.

**Supplementary Figure S11. Full-length western blot images in the figure 3.**

All western blot images are full-length blot images of representative blot data in the figure 3. Dash line box indicates cropped blot image in the figure 3.

**Supplementary Figure S12. Full-length western blot images in the figure 4.**

All western blot images are full-length blot images of representative blot data in the figure 4. Dash line box indicates cropped blot image in the figure 4.

**Supplementary Figure S13. Full-length western blot images in the figure 5.**

All western blot images are full-length blot images of representative blot data in the figure 5. Dash line box indicates cropped blot image in the figure 5.

**Supplementary Figure S14. Full-length western blot images in the figure 6.**

All western blot images are full-length blot images of representative blot data in the figure 6. Dash line box indicates cropped blot image in the figure 6.

**Supplementary Figure S15. Full-length western blot images in the supplementary figure S1b.**

All western blot images are full-length blot images of representative blot data in the supplementary figure S1b. Dash line box indicates cropped blot image in the supplementary figure S1b.

**Supplementary Figure S16. Full-length western blot images in the supplementary figure S3.**

All western blot images are full-length blot images of representative blot data in the supplementary figure S3. Dash line box indicates cropped blot image in the supplementary figure S3.

**Supplementary Figure S17. Full-length western blot images in the supplementary figure S5b.**

All western blot images are full-length blot images of representative blot data in the supplementary figure S5b. Dash line box indicates cropped blot image in the supplementary figure S5b.

**Supplementary Figure S18. Full-length western blot images in the supplementary figure S7.**

All western blot images are full-length blot images of representative blot data in the supplementary figure S7. Dash line box indicates cropped blot image in the supplementary figure S7.

**Supplementary Figure S1**


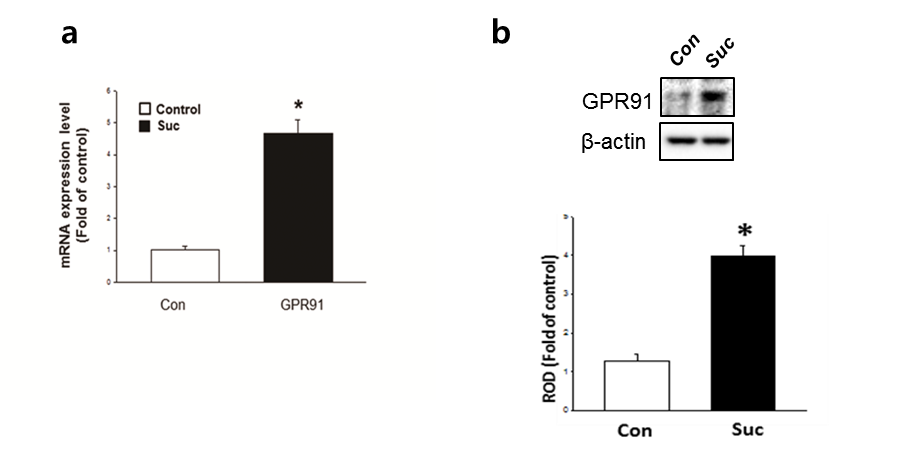


**Supplementary Figure S2**


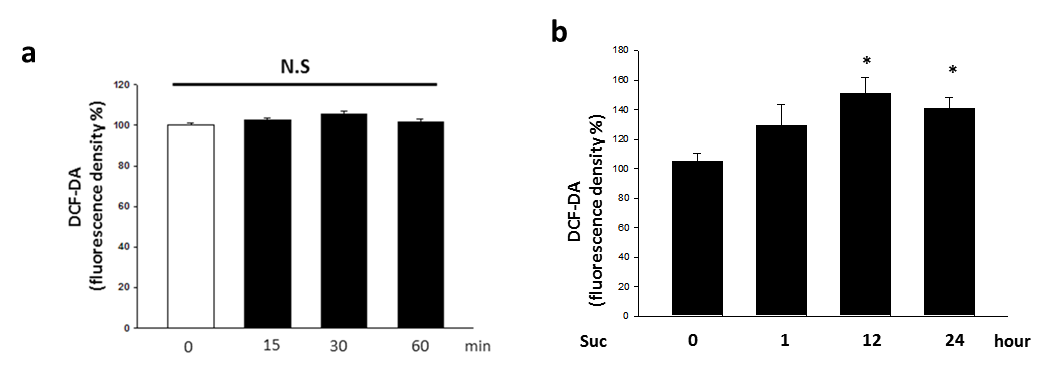


**Supplementary Figure S3**


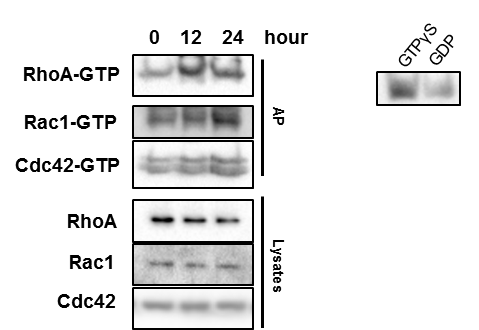


**Supplementary Figure S4**


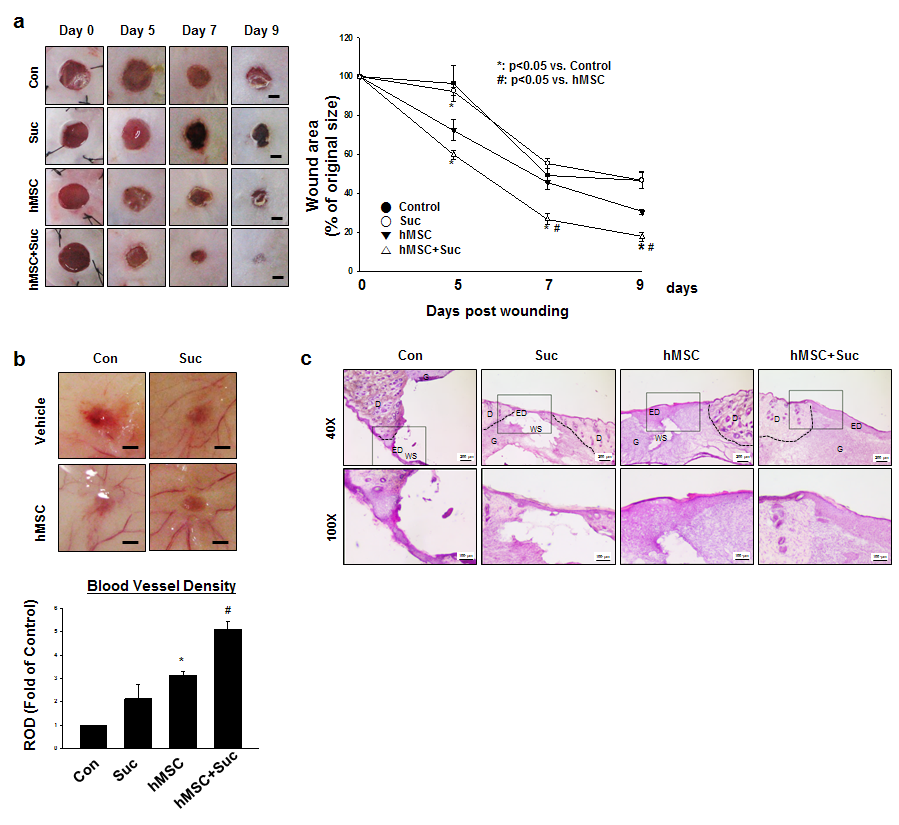


**Supplementary Figure S5**


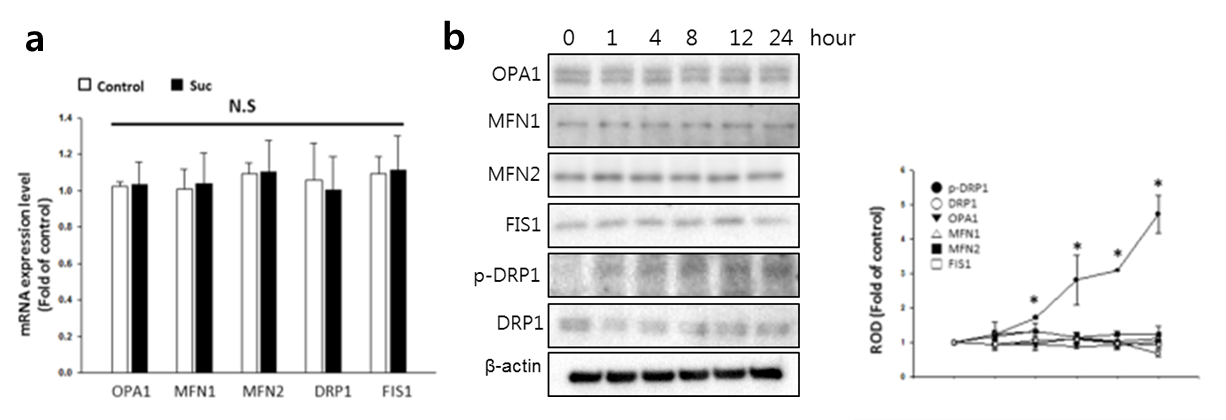


**Supplementary Figure S6**


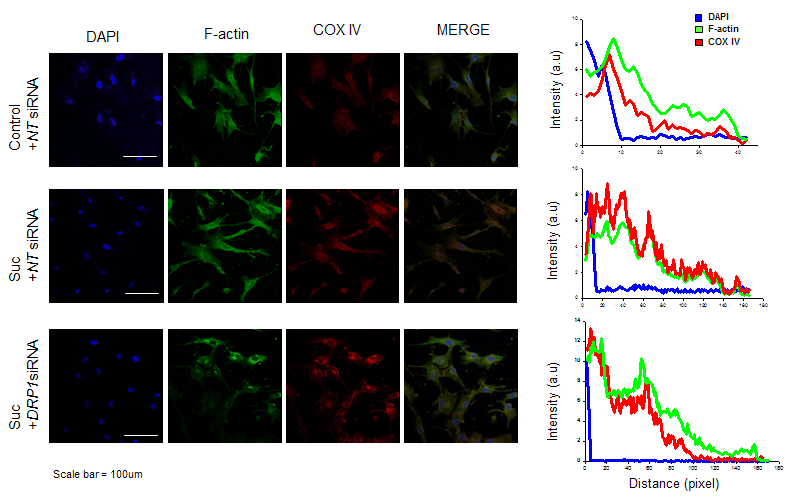


**Supplementary Figure S7**

**
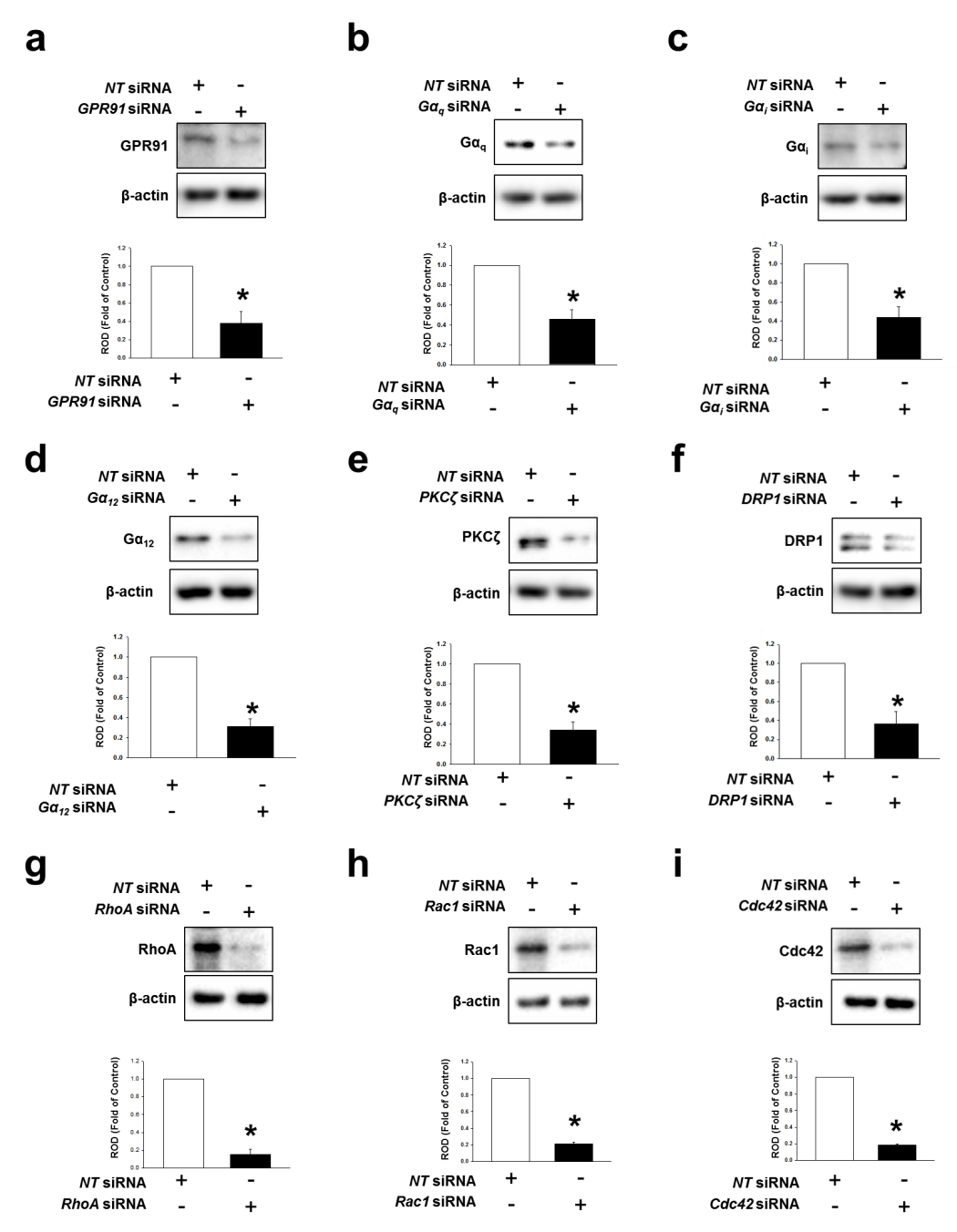
**

**Supplementary Figure S8**

**
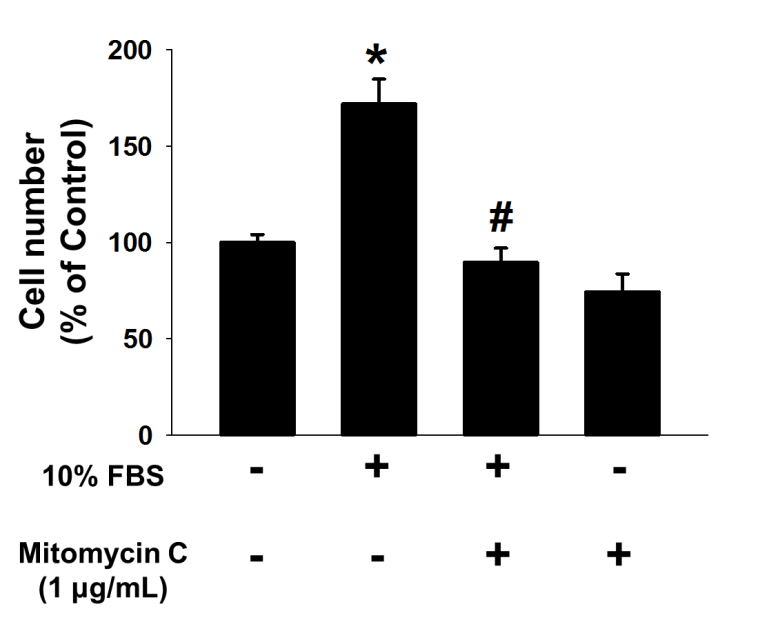
**

**Supplementary Figure S9**

**
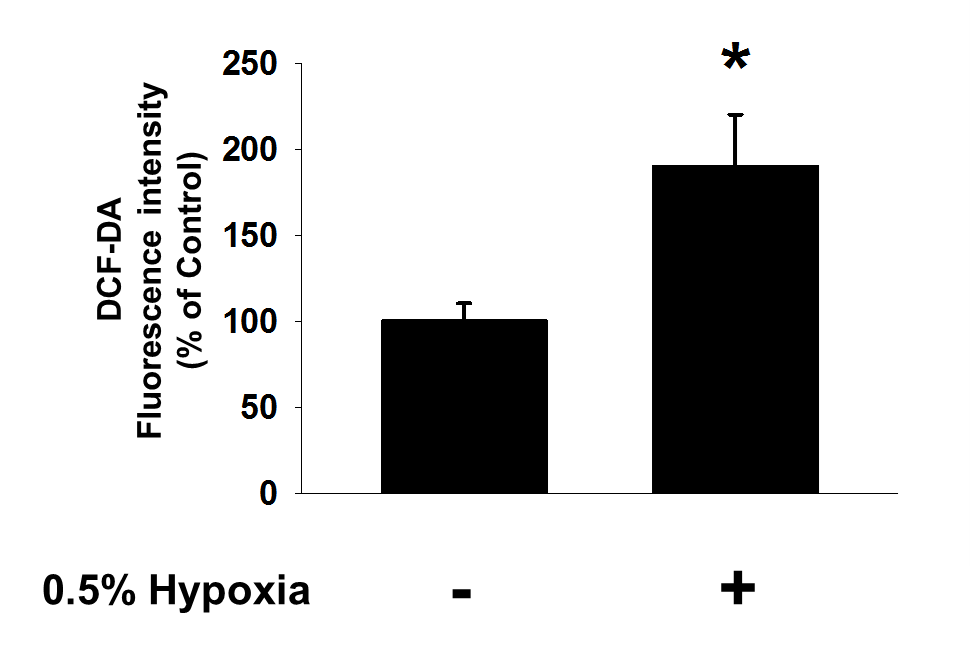
**

**Supplementary Figure S10**

**
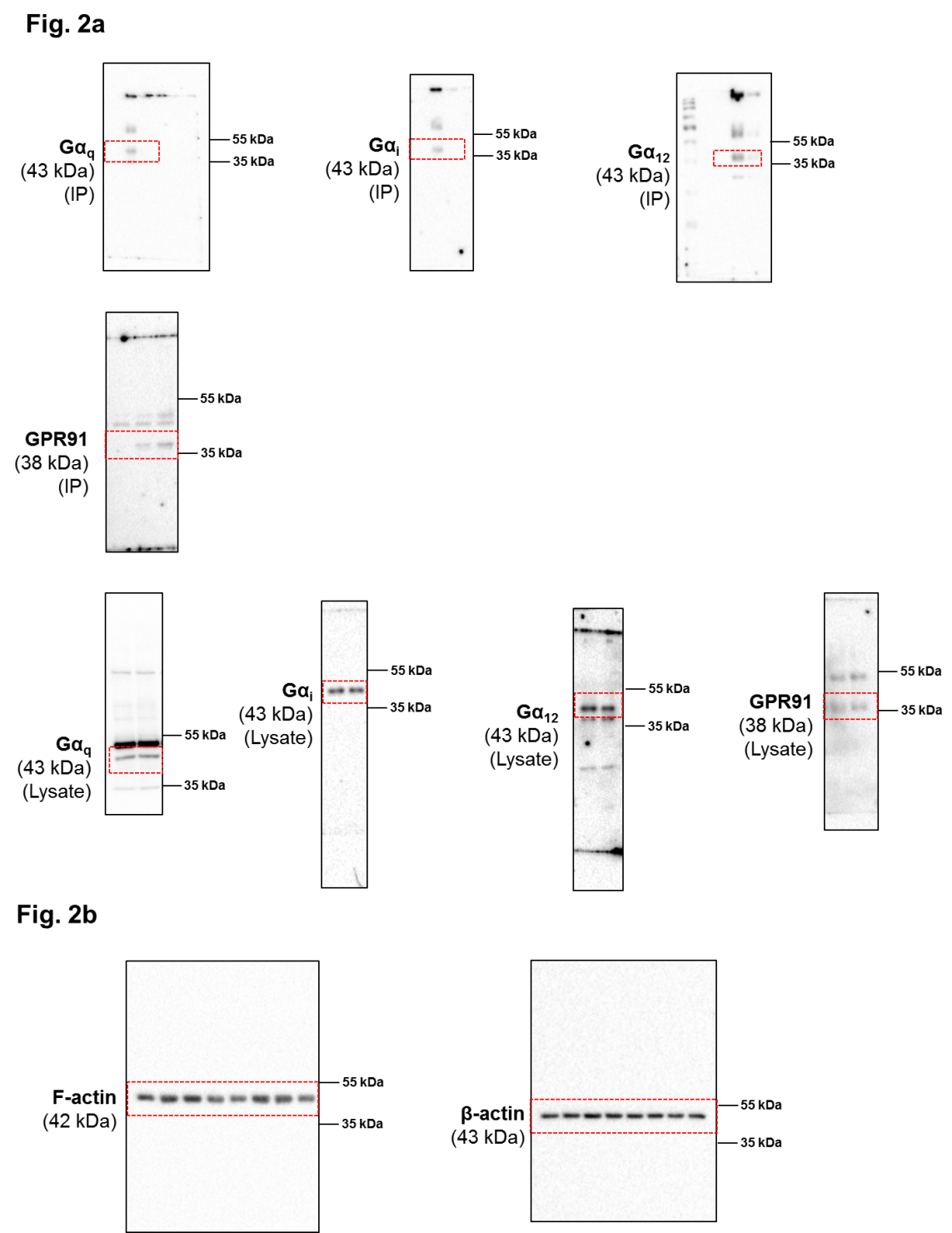
**

**Supplementary Figure S11**

**
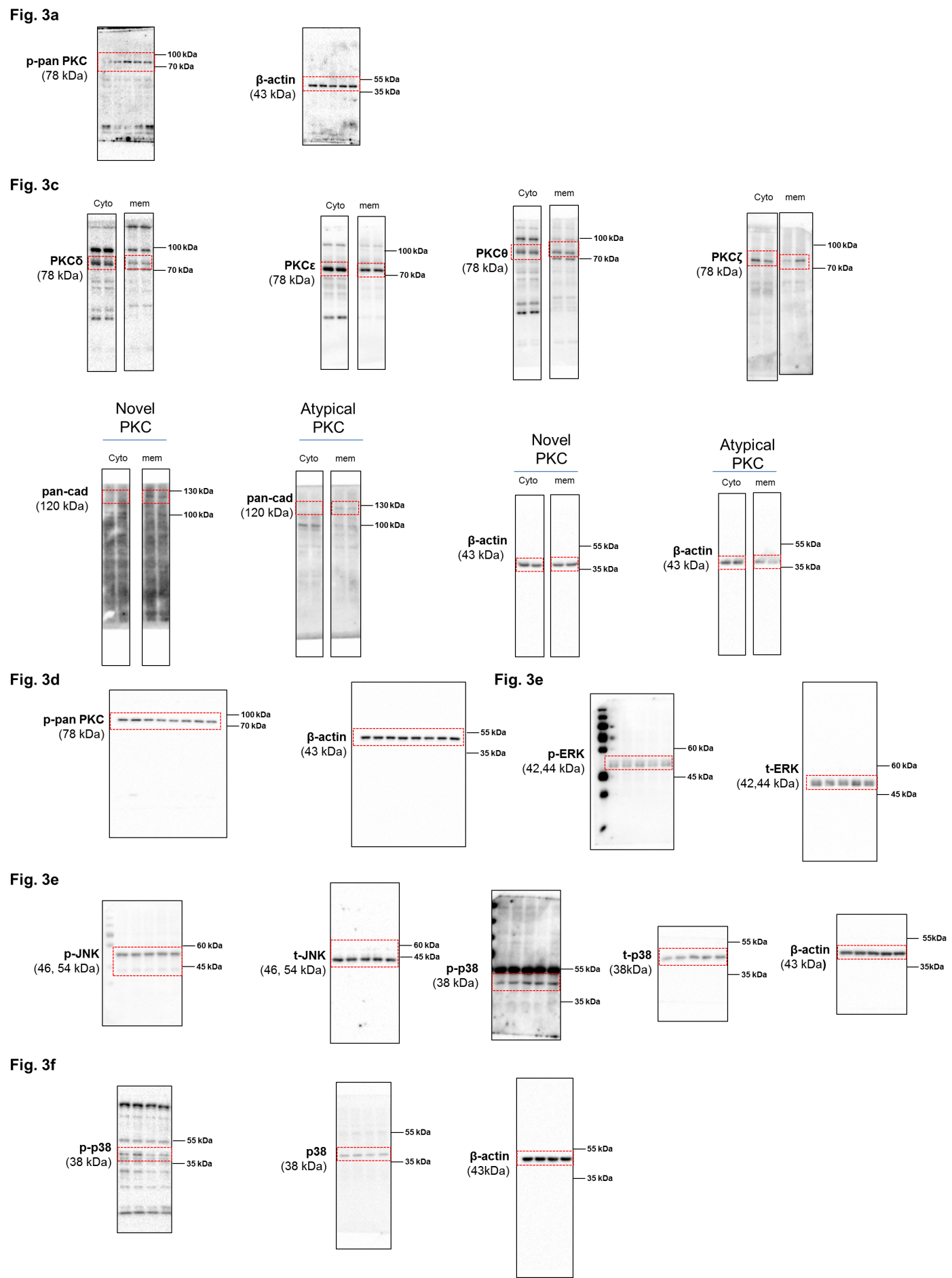
**

**Supplementary Figure S12**

**
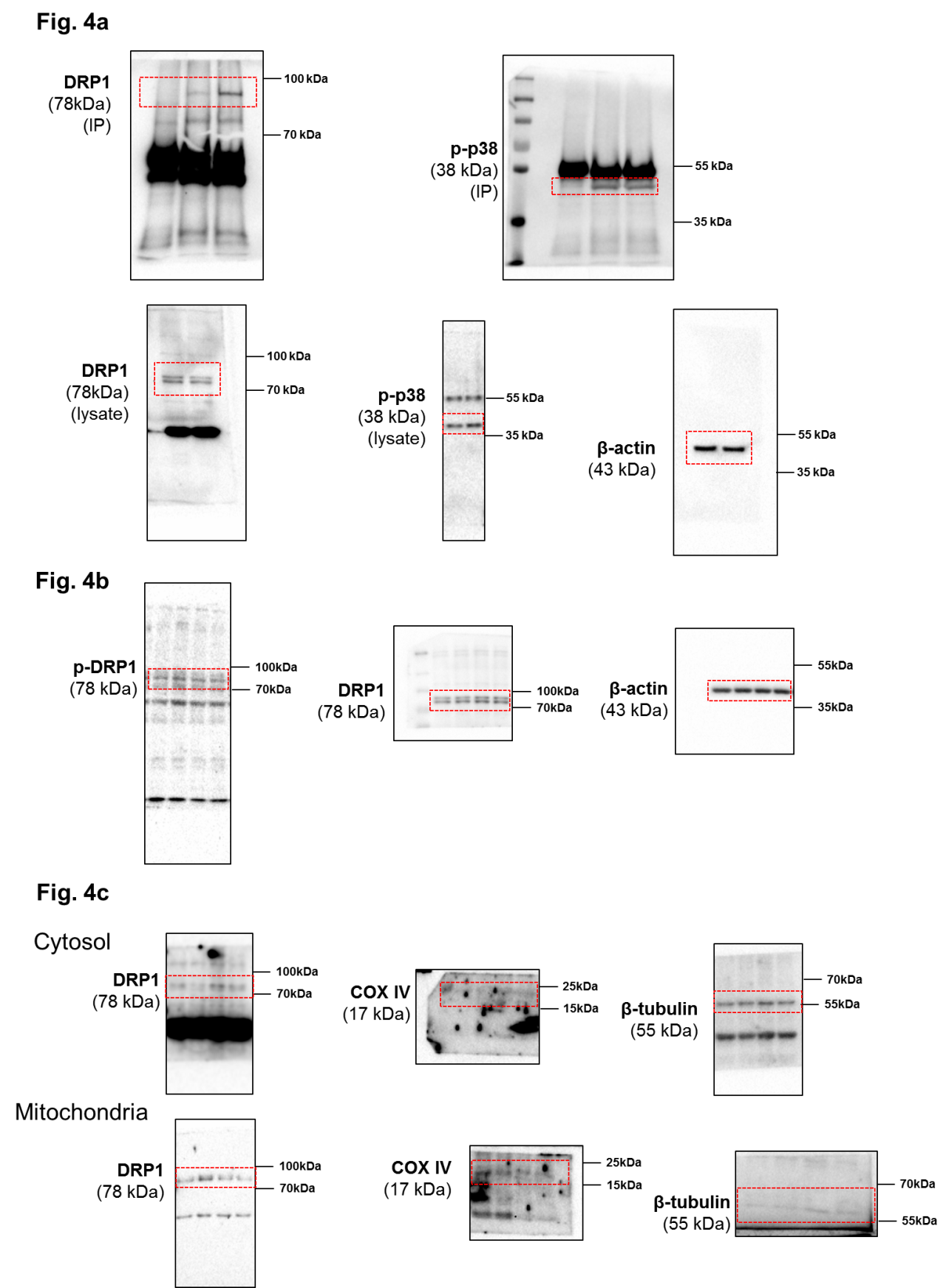
**

**Supplementary Figure S13**

**
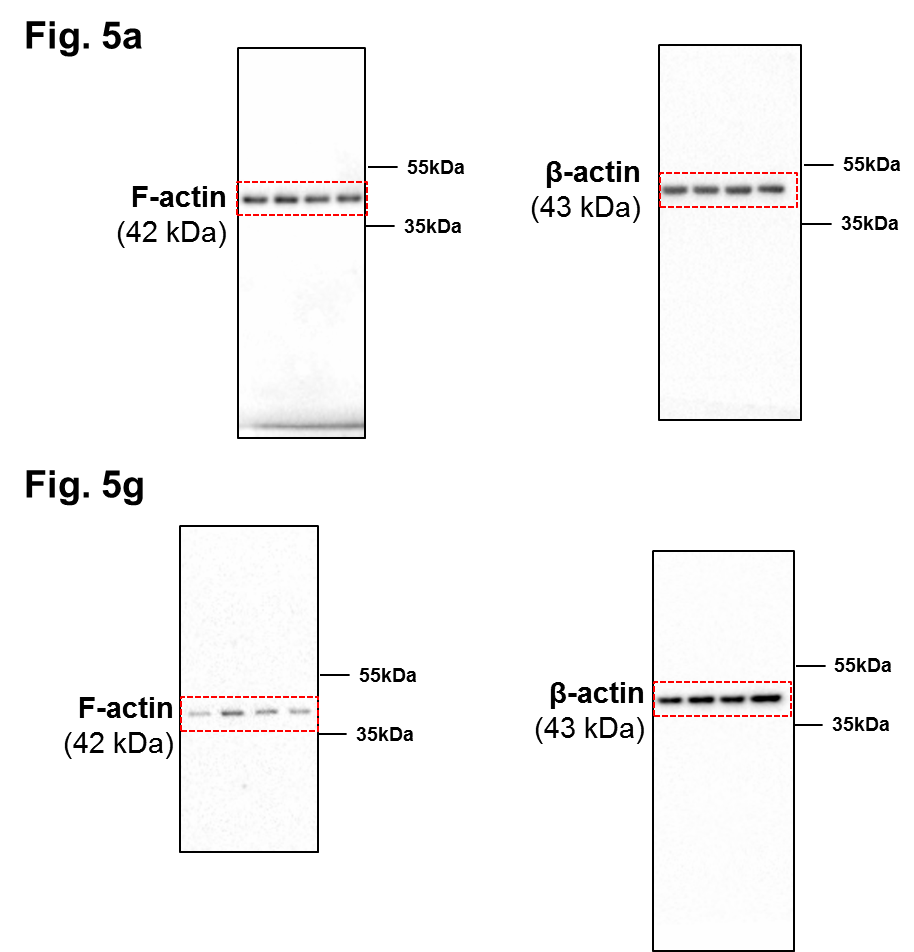
**

**Supplementary Figure S14**

**
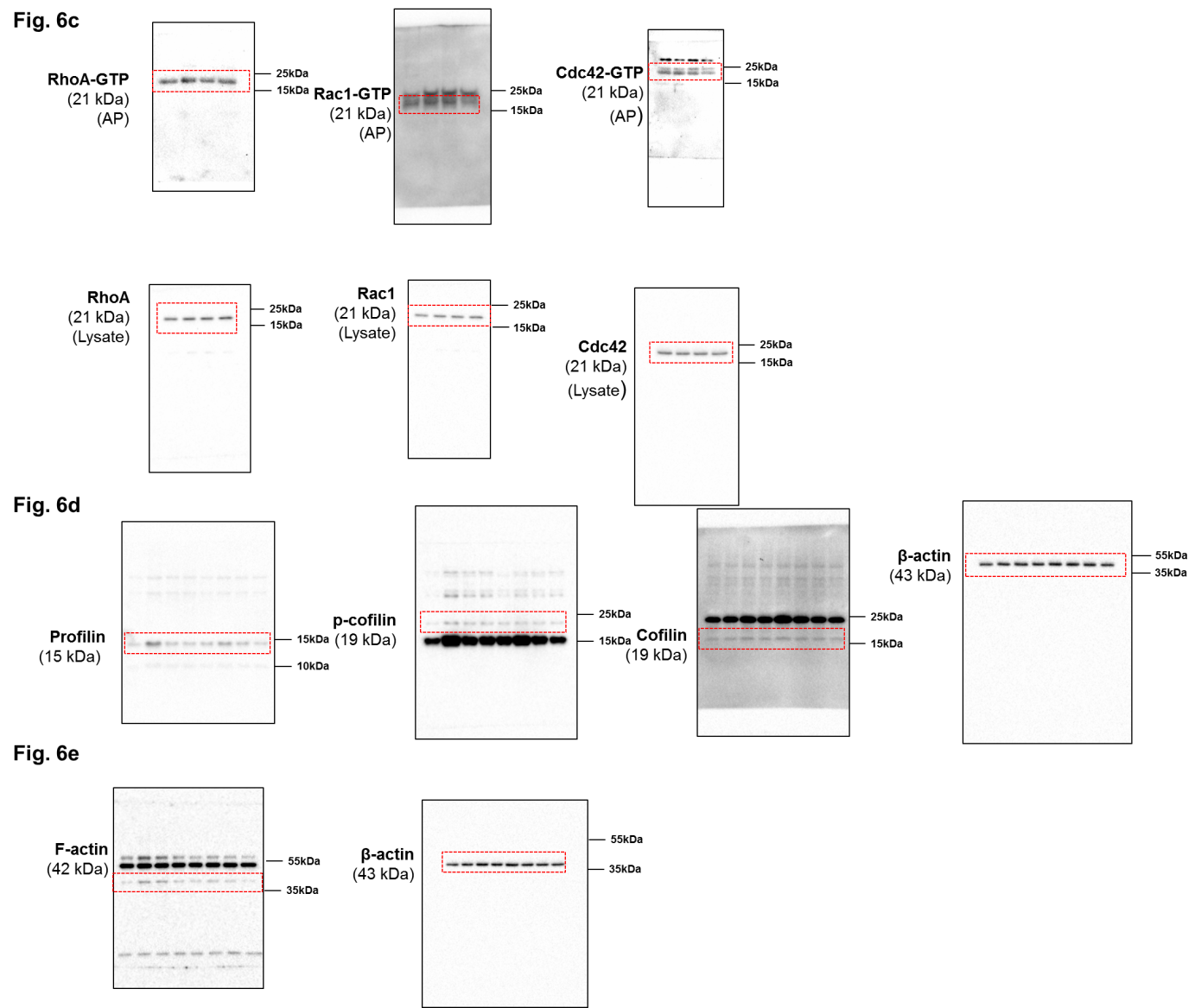
**

**Supplementary Figure S15**

**
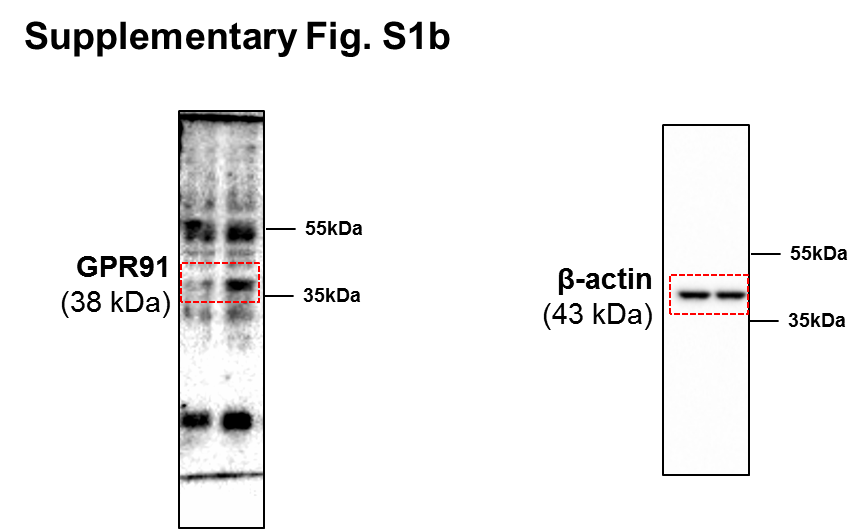
**

**Supplementary Figure S16**

**
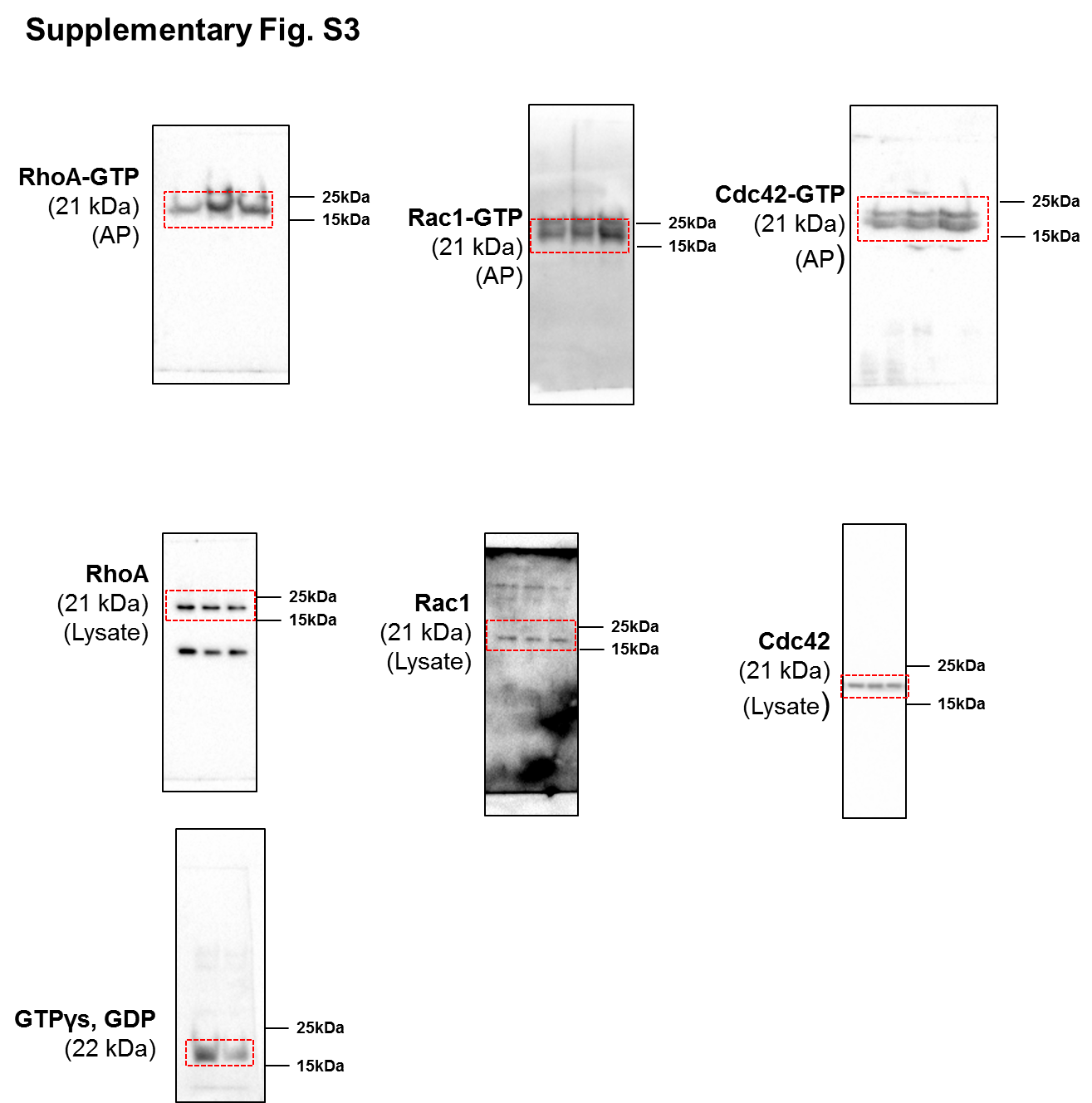
**

**Supplementary Figure S17**

**
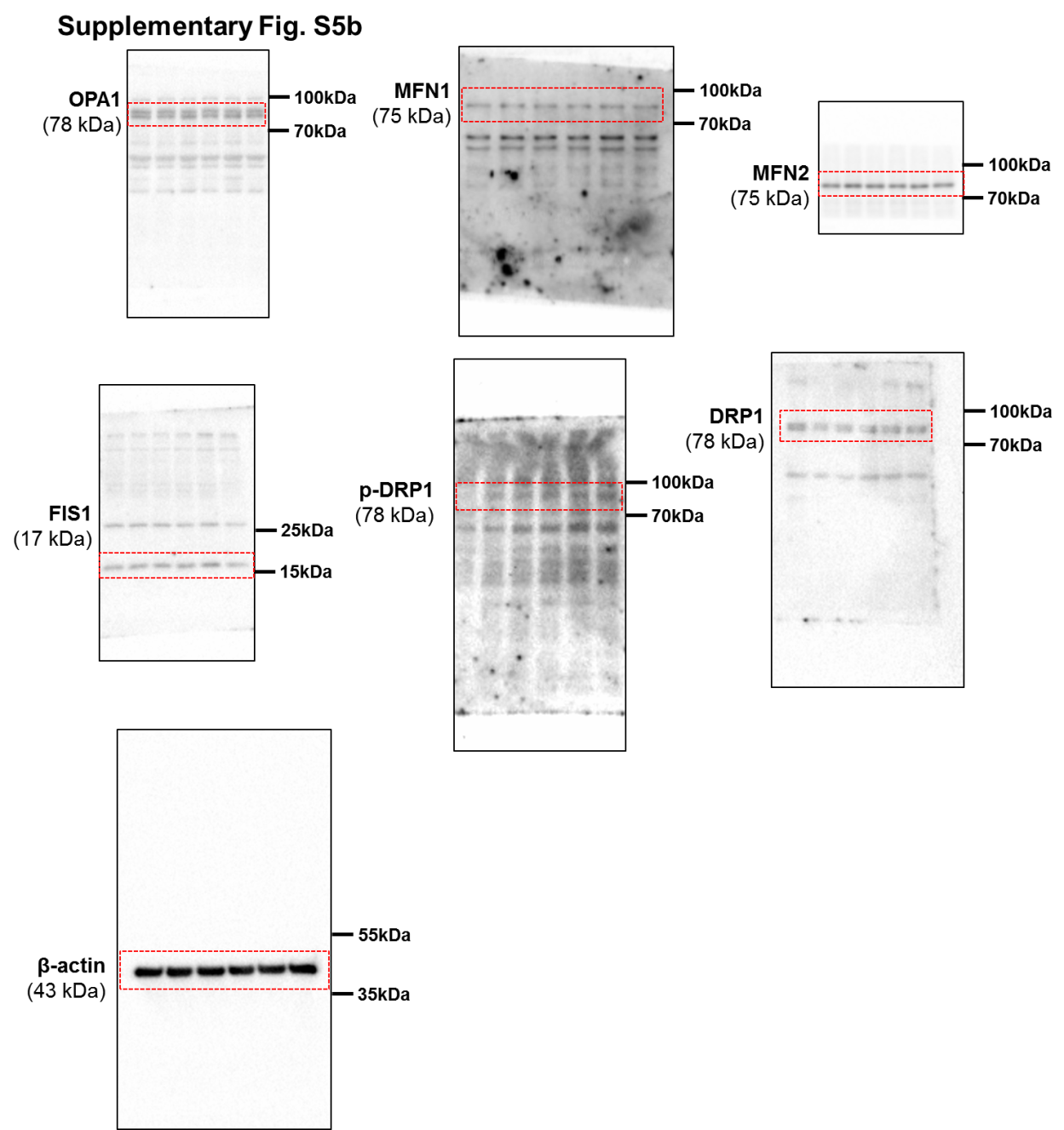
**

**Supplementary Figure S18**


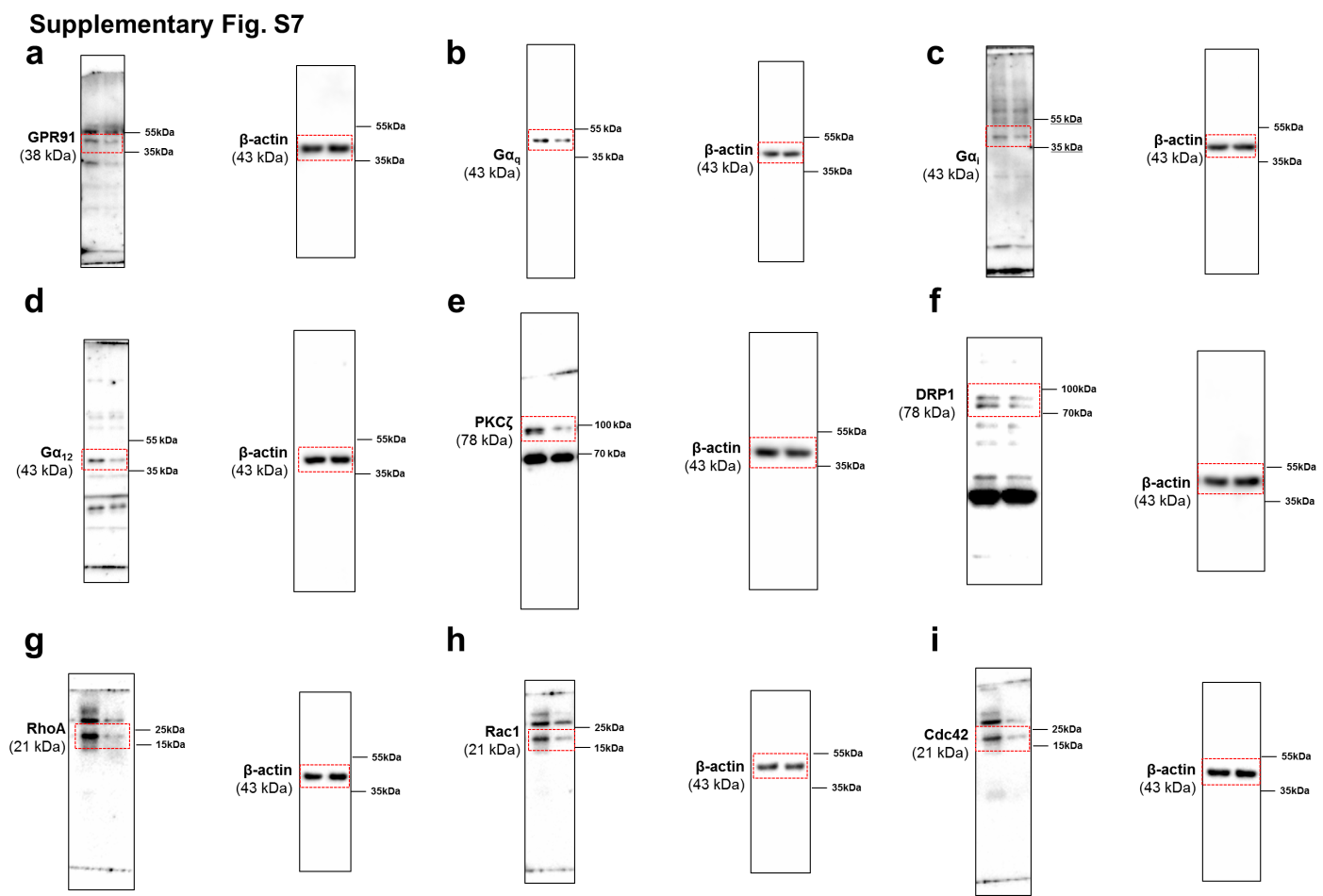


**Supplementary Table S1. Sequences of primers used for RT-PCR**

| **Gene name** | **Identification** | **Sequences (5’-3’)** |
| --- | --- | --- |
| ***GPR91*** | Sense | **CATTGTGACACGGCCTTTGG** |
|  | Antisense | **TGTTTCACAAGCCCCTCACT** |
| ***OPA1*** | Sense | **GCAATGGGATGCAGCTATTT** |
|  | Antisense | **GCAAGATAAGCTGGGTGCTC** |
| ***MFN1*** | Sense | **TGTTTTGGTCGCAAACTCTG** |
|  | Antisense | **CTGTCTGCGTACGTCTTCCA** |
| ***MFN2*** | Sense | **TGTTGGCTCAGTGCTTCATC** |
|  | Antisense | **AAGTCCCTCCTTGTCCCAGT** |
| ***DRP1*** | Sense | **CAGTGTGCCAAAGGCAGTAA** |
|  | Antisense | **GATGAGTCTCCCGGATTTCA** |
| ***FIS1*** | Sense | **CTTGCTGTGTCCAAGTCCAA** |
|  | Antisense | **GCTGAAGGACGAATCTCAGG** |
| ***ACTB*** | Sense | **AACCGCGAGAAGATGACC** |
|  | Antisense | **AGCAGCCGTGGCCATCTC** |

**Reference**

1 Tang, Ho L. *et al.* Vimentin supports mitochondrial morphology and organization. *Biochemical Journal* **410**, 141-146 (2008).
